# Immunogenicity of HIV-1-Based Virus-Like Particles with Increased Incorporation and Stability of Membrane-Bound Env

**DOI:** 10.3390/vaccines9030239

**Published:** 2021-03-10

**Authors:** Christopher A. Gonelli, Hannah A. D. King, Charlene Mackenzie, Secondo Sonza, Rob J. Center, Damian F. J. Purcell

**Affiliations:** 1 Department of Microbiology and Immunology, Peter Doherty Institute for Infection and Immunity, The University of Melbourne, Melbourne, VIC 3000, Australia; chris.gonelli@gmail.com (C.A.G.); hannah.alexandra.king@gmail.com (H.A.D.K.); charlene.mackenzie@mh.org.au (C.M.); secondo.sonza@unimelb.edu.au (S.S.); rob.center@burnet.edu.au (R.J.C.); 2 Viral Entry and Vaccines Laboratory, Disease Elimination, Burnet Institute, Melbourne, VIC 3004, Australia

**Keywords:** HIV-1, virus-like particle, VLP, mature form, Env incorporation, SOSIP

## Abstract

An optimal prophylactic vaccine to prevent human immunodeficiency virus (HIV-1) transmission should elicit protective antibody responses against the HIV-1 envelope glycoprotein (Env). Replication-incompetent HIV-1 virus-like particles (VLPs) offer the opportunity to present virion-associated Env with a native-like structure during vaccination that closely resembles that encountered on infectious virus. Here, we optimized the incorporation of Env into previously designed mature-form VLPs (mVLPs) and assessed their immunogenicity in mice. The incorporation of Env into mVLPs was increased by replacing the Env transmembrane and cytoplasmic tail domains with those of influenza haemagglutinin (HA-TMCT). Furthermore, Env was stabilized on the VLP surface by introducing an interchain disulfide and proline substitution (SOSIP) mutations typically employed to stabilize soluble Env trimers. The resulting mVLPs efficiently presented neutralizing antibody epitopes while minimizing exposure of non-neutralizing antibody sites. Vaccination of mice with mVLPs elicited a broader range of Env-specific antibody isotypes than Env presented on immature VLPs or extracellular vesicles. The mVLPs bearing HA-TMCT-modified Env consistently induced anti-Env antibody responses that mediated modest neutralization activity. These mVLPs are potentially useful immunogens for eliciting neutralizing antibody responses that target native Env epitopes on infectious HIV-1 virions.

## 1. Introduction

Vaccination with live attenuated viruses has been a highly successful public health intervention as exemplified by the control of poliomyelitis and eradication of smallpox, both of which previously caused substantial mortality and morbidity globally. A similar vaccine strategy for human immunodeficiency virus (HIV-1) is not feasible given its highly error-prone replication cycle and ability to form recombinant genomic sequences that collectively facilitate reversion to pathogenic virus. For this reason, much research has been conducted into developing subunit vaccines using components of the virus, particularly the viral envelope glycoprotein (Env), which can elicit neutralizing antibodies capable of preventing HIV-1 infection [1,2]. The poor immunogenicity of Env has caused difficulties in consistently eliciting such antibodies via existing vaccination strategies—it has an extensive host-derived glycan shield preventing antibody binding to the underlying protein, thus dampening immune responses [3,4,5,6,7], and can be structurally flexible, exposing sites that elicit immunodominant non-neutralizing responses [8,9,10,11,12]. Additionally, the elicitation of broadly protective responses is complicated by the high degree of amino acid sequence variability between and within circulating virus clades [13,14,15].

Virus-like particle (VLP) immunogens have the advantage over conventional recombinant protein vaccines of larger size and repetitive display of antigenic epitopes, and therefore are better at stimulating the innate and adaptive arms of the immune system [16,17,18,19]. Indeed, the clinical utility of VLP-based immunogens has been exemplified by widely approved VLP vaccines against hepatitis B and human papilloma virus [20,21,22,23]. However, for membrane-bound Env presented on HIV-1 VLPs, the labile interactions that maintain gp120-gp41 heterodimers within Env trimers [24,25] can result in shedding of gp120 from the surface of viral particles [26,27]. This is further compounded by the inherently low incorporation of Env into HIV-1 virions [28,29,30,31] compared to other enveloped mammalian viruses [32]. However, it has been shown that chimeric HIV-1 Env bearing the transmembrane (TM) and cytoplasmic tail (CT) domains from highly surface-expressed non-HIV-1 envelope glycoproteins are efficiently incorporated into HIV-1 VLPs [33]. This relies on a passive incorporation of Env into the viral particle, which is routinely used in the generation of lentiviral vectors expressing non-HIV-1 surface glycoproteins, such as vesicular stomatitis virus and murine leukemia virus (MuLV) glycoproteins [34].

An alternative tactic for high-surface-density, particulate presentation of Env is the use of self-assembling ferritin nanoparticle systems [35,36,37,38,39,40,41] or liposomes bearing an array of Env trimers [42,43,44,45]. However, these synthetic-particle approaches are only applicable to a limited number of Env strains as they rely on the soluble Env efficiently forming native-like trimers, which has only been demonstrated for a minority of Env strains from certain clades [46,47,48]. In contrast, the presence of the TM domain in membrane-bound Env presented on VLPs is likely to facilitate a compact, trimeric Env conformation for a broader range of Env strains [49]. Therefore, the presentation of Env on a VLP immunogen is not limited to specific Env strains, which is advantageous given the high sequence variability of Env in circulating isolates [13,14,15]. Additionally, in nonhuman primate vaccinations with soluble trimers, analysis of the polyclonal antibody response demonstrated the exposed trimer base is heavily targeted [50]. The membrane-bound Env on VLP immunogens prevents this immunodominant neo-epitope from being targeted through steric occlusion.

Vaccination with nativelike trimer immunogens resembling the functional Env on viral particles has been shown to elicit more potent serum antibodies that mediate viral neutralization than heterogeneous populations of non-native Envs [51]. The functional trimeric species are indeed more effective at presenting bNAb epitopes than other nonfunctional Env multimers or monomers [52]. Consequently, the uniform presentation of trimeric Env on the surface of VLP immunogens is desirable for the generation of useful antibody responses. VLPs, as is the case for wild-type HIV-1 virions, present multiple conformers of Env, including trimeric and monomeric gp120-gp41 heterodimers, as well as gp41 subunits from which gp120 has dissociated, on their surface [9]. While methods involving enzymatic digestions to remove the forms of Env that bind non-neutralizing antibodies are effective at occluding or eliminating these epitopes [52,53,54,55], they introduce additional processing steps on top of the already laborious task of producing VLPs for large-scale vaccination [56].

We previously described a vector expressing mature (proteolytically cleaved Gag core) HIV-1 VLPs [57] predicted to better trigger B-cell-receptor cross-linking, since core maturation facilitates surface Env clustering on virions [58,59]. The noninfectious mature VLP (mVLP) expression cassette lacked HIV-1 promoters, enzyme functions needed for host genome integration, and accessory proteins Vif, Vpr, and Nef [57]. We determined the minimal Gag and protease domain sequences required for efficient particle expression and maturation, and generated VLPs with similar morphology, size, and function (in terms of target-cell fusion) when compared to fully infectious, mature virions.

This paper describes the immunogenicity of these mVLPs to immature (Gag-only core) VLPs, as well as the introduction of modifications to enhance incorporation and stabilization of Env on mVLPs. The immunogenicity of these VLPs was examined in mice, with the aim of inducing humoral responses capable of blocking virus infection. The Env expression cassette within a single HIV-1 VLP-producing vector developed previously [57] was modified within the TM and CT domains to increase Env incorporation into VLPs. Additionally, the introduction of a gp120-gp41 disulfide linkage in combination with a gp41 I559P mutation (SOSIP) stabilized the Env conformation and improved its antigenicity, as demonstrated by increased binding to bNAbs, particularly those targeting quaternary epitopes, and reduced interaction with non-neutralizing antibodies. Vaccination of mice with mVLPs bearing stabilized Env elicited a broad range of Env-specific antibody isotypes, as well as modest tier 1 neutralizing activity when VLPs with a higher surface density of Env were utilized.

## 2. Materials and Methods

### 2.1. Cells, Enzymes, and Oligonucleotides

HEK 293T cells were maintained in high-glucose DMEM (Media Preparation Unit (MPU), The University of Melbourne, Australia) supplemented with GlutaMAX™-I and 10% (*v*/*v*) heat-inactivated fetal bovine serum (HI-FBS) (both components Thermo Fisher Scientific, Waltham, MA, USA) (growth medium) and transiently transfected using Lipofectamine 2000 (Thermo Fisher Scientific) according to the manufacturer’s instructions. Suspension CEM.NKR CCR5+ cells were maintained in RPMI-1640 (MPU) medium supplemented with GlutaMAX™-I and 10% (*v*/*v*) HI-FBS. Expi293F cells were cultured in baffled Erlenmeyer flasks with Gibco Expi293 Expression Medium and transiently transfected using ExpiFectamine 293 (Thermo Fisher Scientific) according to manufacturer’s instructions. All cell lines were routinely screened and confirmed negative for mycoplasma contamination using the PlasmoTest™ Mycoplasma Detection Kit (InvivoGen, San Diego, CA, USA). All the PCRs were performed using Phusion^®^ DNA polymerase (NEB, Ipswich, MA, USA), DNA plasmid ligations were performed using T4 DNA Ligase (Thermo Fisher Scientific), and all other enzymes used for plasmid construction were obtained from NEB. DNA fragments generated de novo were synthesized by Integrated DNA Technologies (Coralville, IA, USA). The oligonucleotides and chemicals were purchased from MilliporeSigma (Burlington, MA, USA), unless otherwise specified.

### 2.2. Monoclonal and Polyclonal Antibodies

The monoclonal antibodies (mAbs) 17b [60], 35O22 [61], 447-52D [62], F105 [63], F240 [64], PGT121 [65], and Z13e1 [66] were obtained from the National Institutes of Health (NIH) HIV Reagent Program (HRP). Anti-Env mAbs 2F5, 2G12, 4E10, PG9, and PG16 were purchased from Polymun (Vienna, Austria). Anti-p24 mAb BC1071 and sheep polyclonal antibodies anti-p24 (D7320) and anti-gp120 (D7324) were purchased from Aalto Bio Reagents (Dublin, Ireland). Anti-GAPDH (14C10) rabbit mAb was purchased from Cell Signaling Technology (Danvers, MA, USA).

Heavy- and light-chain expression plasmids for 10E8, PGT151, and CAP256-VRC26.06 (synthesized from published protein sequences [67,68,69], respectively) were kindly obtained from Pantelis Poumbourios (Burnet Institute, Melbourne, Australia), and VRC01 expression plasmids were obtained from the NIH HRP [70]. Antibody heavy and light chains were coexpressed in Expi293F cells and purified using Protein G Agarose Fast Flow (MilliporeSigma) according to the manufacturer’s instructions.

### 2.3. Plasmids

Plasmids to express SOSIP-stabilized gp140 encoded by the Env from NL(AD8) [71] (herein referred to as AD8 strain) were generated from pN1-AD8-140 [72] by introducing A501C, I559P, and T605C substitutions (HXB-2 numbering used here and throughout [73]), reverting the nonfunctional cleavage site to its wild-type R^508^EKR, and truncating the membrane proximal external region (MPER) from residue 664. Additionally, a C-terminal 6xHis tag (ASGSGHHHHHH) or D7324 epitope tag (GSAPTKAKRRVVQREKR) was added after residue 664 to generate pN1-AD8140SOSIP664-6H or pN1-AD8SOSIP664-D7, respectively.

**Figure 1:**
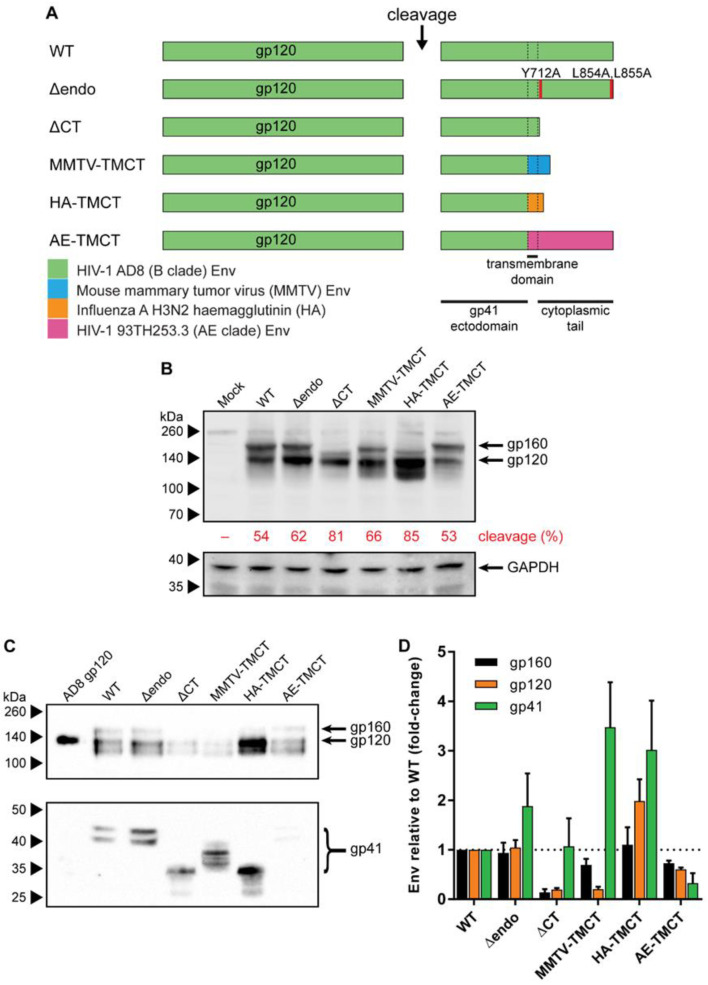
Genetic organization, expression, and VLP incorporation of Env with TM and CT domain modifications. (**A**) Schematic representation of TM- and CT-domain-modified AD8 gp160 sequences compared to the wild-type (WT) sequence. Env CT motifs associated with endocytosis were removed (Δendo) by introducing Y712A, L854A, and L855A amino-acid substitutions (red shading). The CT was completely removed (ΔCT) by truncating Env immediately C-terminal of the TM. The HIV-1 AD8 TM and CT were replaced with the equivalent TM and CT sequences from mouse mammary tumor virus (MMTV-TMCT), influenza A (IFA) subtype H3N2 haemagglutinin (HA-TMCT), or HIV-1 93TH253.3 strain (AE clade) Env (AE-TMCT). Dashed lines indicate boundaries between gp41 ectodomain, TM, and CT. Shading represents source of TM/CT sequence in mutants according to key in lower left. (**B**) Representative anti-gp120 (upper panel, probed with D7324) and anti-GAPDH (lower panel, probed with 14C10) Western blot of 293T cell lysate following transfection with an empty vector (Mock) or vectors expressing the gp160 sequences described in (**A**). The percent of cleaved Env (gp120) relative to gp160 expression was calculated for Env sequence by densitometric analysis. (**C**) Representative anti-gp120 (D7324) and anti-gp41 (2F5) Western blot of Env-deficient mVLPs (mVLPΔenv) pseudotyped with wild-type AD8 gp160 (WT) and various TM- and CT-modified gp160 as described in (**A**). The 200ng recombinant AD8 gp120 is shown as reference. VLP sample loading was equalized by p24 ELISA. For (**B**,**C**), proteins were resolved by 8–16% SDS-PAGE under reducing conditions. Protein sizes were indicated by the Spectra Multicolor Broad Range Protein Ladder (Thermo Fisher Scientific) and shown on the left. The position of gp160, gp120, and gp41 bands are indicated. (**D**) Western blot densitometric analysis on TM- and CT-modified Env gp160, gp120, and gp41 incorporation into mVLPs as fold-change relative to mVLPs pseudotyped with WT gp160. Values shown are the mean and SEM of 3 independent experiments.

**Figure 2:**
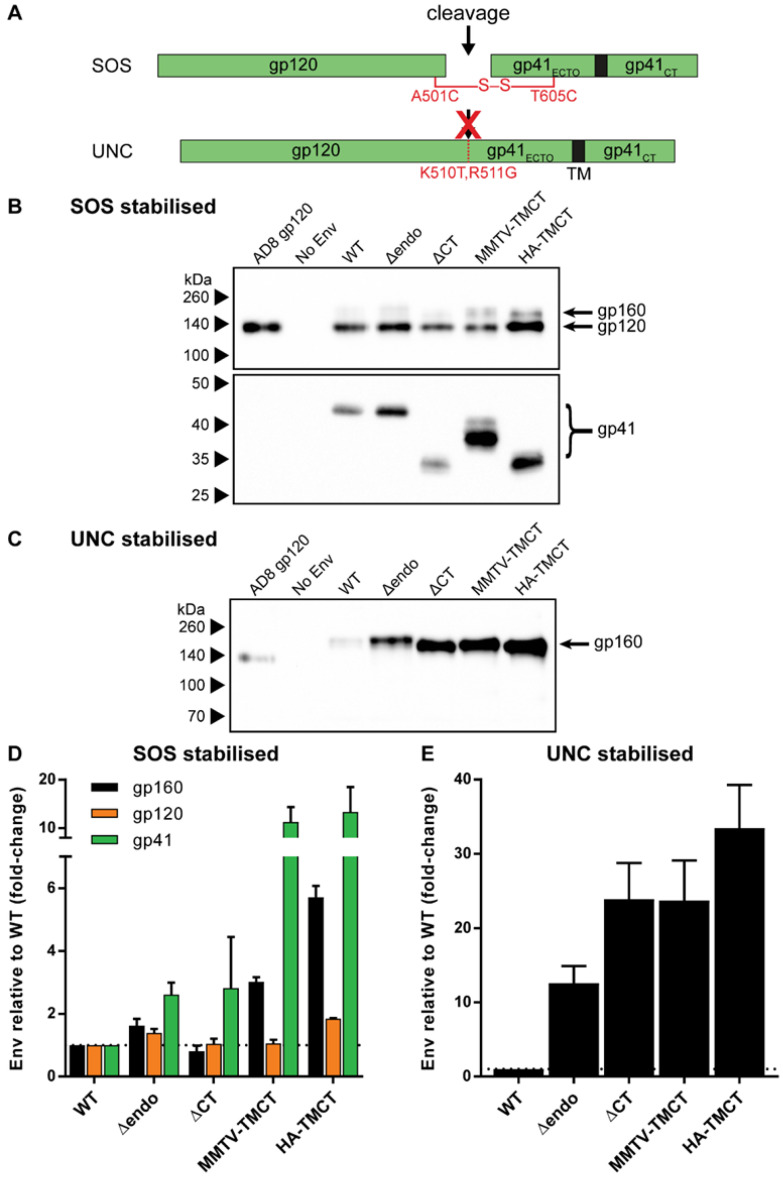
Incorporation of stabilized Env with TM and CT domain modifications into mVLPs. (**A**) Schematic representation of covalent stabilizing mutations between gp120 and gp41 domains used in combination with TM and CT modifications detailed in Figure 1A. Env was stabilized via an intermolecular disulfide bond (SOS) or by removing a furin-like cellular protease recognition site (UNC). (**B**) Representative anti-gp120 (D7324, upper panel) and gp41 (2F5, lower panel) Western blot of mVLPΔenv pseudotyped with an empty vector (no Env), wild-type AD8 gp160 (WT), or various TM- and CT-modified gp160 (as detailed in Figure 1A) that also contain the SOS stabilization modification (see (**A**)). (**C**) Representative anti-gp120 (D7324) Western blot of mVLPΔenv pseudotyped with an empty vector (no Env), wild-type AD8 gp160 (WT), or various TM- and CT-modified gp160 that also contain the UNC stabilization modification (see (**A**)). For both (**B**) and (**C**), 200 ng AD8 gp120 was loaded, VLP sample loading was equalized by p24 ELISA (data not shown), and proteins were resolved by 8–16% SDS-PAGE under reducing conditions. Protein sizes were indicated by the Spectra Multicolor Broad Range Protein Ladder and are shown on the left. The position of gp160, gp120, and gp41 bands used for subsequent densitometry analysis is indicated. Western blot densitometric analysis on TM- and CT-modified (**D**) SOS-stabilized and (**E**) UNC-stabilized Env gp160, gp120, and gp41 incorporation into mVLPs as fold-change relative to mVLPs pseudotyped with Env expressing a wild-type HIV-1 TM and CT (WT). Values shown are the mean and SEM of 3 independent experiments.

The plasmid pCMV-AD8 [74], which expresses wild-type (WT) AD8 Env, was used as the template vector for TM and CT modifications (illustrated summary in Figure 1A). The Δendo Env was generated by introducing Y712A, L854A, and L855A substitutions. The ΔCT Env was created by introducing a premature termination codon at residue 708. The MMTV-TMCT and HA-TMCT Env plasmids were generated by substituting the Env TM and CT with sequences from mouse mammary tumor virus (MMTV) envelope glycoprotein (GenBank accession# AF228552.1) and influenza virus A (IFA) haemagglutinin (HA) (accession# DQ227430.1), respectively. In both cases, the TM and CT substitutions were inserted into the HIV-1 Env sequence immediately N-terminal to the HIV-1 Env TM such that the second Rev coding exon reading frame and splice acceptors would not be interrupted, which was critical given the Env expression plasmid relies on Rev to mediate Env mRNA nuclear export [75]. DNA fragments encoding the MMTV and HA TM and CT were synthesized according to their original nucleotide sequences with silent mutations to abolish potential splice donor and acceptor sequences, as predicted by the Human Splicing Finder bioinformatic tool [76]. The AE-TMCT Env expression plasmid was produced by replacing the AD8 *env* TM and CT with the HIV-1 93TH253.3 strain *env* TM/CT sequence amplified from p93TH253.3 [77]. The WT and all TM/CT modified Env expression plasmids (except AE-TMCT) were further modified by introduction of a disulfide bond between the gp120 and gp41 domains via A501C and T605C substitutions (SOS) or mutation of the cellular protease cleavage site between the gp120 and gp41 domains via K510T and R511G substitutions (UNC) (illustrated in Figure 2A). The SOS HA-TMCT Env expression plasmid was additionally modified via an I559P substitution to generate the SOSIP HA-TMCT Env expression plasmid.

For Env expression vectors bearing WT TM and CT sequences, flexible linker sequences of 10 (L10) or 15 (L15) amino acids (composed of repeats of SGGGG) were inserted between residues 511 and 512 of the uncleaved Env expression plasmid (UNC) and SOS-modified uncleaved Env plasmid (UNC SOS) by PCR mutagenesis. The WT SOS, UNC SOS, L10 SOS, and L15 SOS plasmids were also further modified with an I559P substitution to yield SOSIP-modified Env expression plasmids. D7324-tagged gp140 expression plasmids were generated for the WT, UNC, and each linker, SOS, and SOSIP vector by taking the KpnI/HindIII fragment from each vector and using it to replace the equivalent fragment within pN1-AD8SOSIP664-D7.

VLP-expression vectors producing immature VLPs (iVLPs) (pCMV-AEp55B) or mVLPs (pCMV-AEB.NCΔRT) bearing WT AD8 Env, and Env-deficient mVLPs (mVLP Δenv) (pCMV-AEΔB.NCΔRT) were previously described [57]. A non-particle-producing control VLP vector (ΔVLP) was generated by introducing nonsense mutations at the Gag start codon and all potential initiation codons within p17 and at the beginning of p24 (M1X, M31X, M142X) and excising part of the p24 domain (illustrated summary in Figure S3A). VLP vectors expressing Env with modified TM/CT sequences and/or stabilizing mutations (SOS or SOSIP) were all assembled by cloning the KpnI/BlpI fragment from their respective gp160 expression vectors into the same sites in the parental VLP vector (mVLP, iVLP, or ΔVLP).

The Env-deficient NL4-3 virus expression plasmid, pDRNL4-3Δenv, was generated from pDRNL4-3 (described previously in [75]) by replacing the *env* sequence with a prematurely terminating *env* sequence (nt 7037–7619, derived from pHIS-HIV-B [78]) such that a single-round infectious virus could be generated when pseudotyped with an envelope glycoprotein. This plasmid was also rendered Vpr-deficient (pDRNL4-3ΔenvR-) by introducing a frameshift at amino acid residue 26 within *vpr* by cutting and end-filling the AflII site (nt 5180).

### 2.4. Recombinant Env

Recombinant, monomeric AD8 gp120 and trimeric, uncleaved AD8 gp140 (AD8 UNC gp140) were produced as described previously [57,72,79]. Trimeric, His-tagged SOSIP-stabilized AD8 gp140 (AD8 SOSIP gp140) was expressed in Expi293F cells cotransfected with pN1-AD8140SOSIP-6H and a human furin expression plasmid (pcDNA3.1 furin) (kindly provided by Mark Hogarth (Burnet Institute) [80]) at an Env-to-Furin DNA mass ratio of 5:1. Culture supernatant was dialyzed into binding buffer (300 mM NaCl, 20 mM imidazole in phosphate-buffered saline [PBS]), applied to a HiTrap IMAC HP column (GE Healthcare Life Sciences, Little Chalfont, UK), and after washing, Env was eluted with a linear gradient of increasing imidazole up to 500 mM. Trimeric Env-containing elution fractions were concentrated, and buffer-exchanged into PBS. Size-exclusion chromatography (SEC) on a HiLoad Superdex 200 pg 16/600 column (GE Healthcare) run at 1 mL/min in PBS was then used to further purify trimeric gp140. D7324-tagged AD8 SOSIP gp140 was expressed as above, except the plasmid pN1-AD8140SOSIP664-D7 was used. The purification strategy was adapted from previously published protocols [81]. Env was purified from culture supernatant by lentil lectin affinity chromatography as described previously [79], except Triton X-100 was not used and the elution buffer contained 1M methyl α-D-mannopyranoside. The Env-containing elution fractions were buffer-exchanged into 20 mM Tris (pH 8.0) containing 100 mM NaCl and applied to a HiTrap Q FF anion-exchange (AEX) column (GE Healthcare) in flow-through mode before being buffer-exchanged into 100 mM phosphate buffer (pH 7.0) containing 2 M (NH_4_)_2_SO_4_ and loaded onto a HiTrap Phenyl HP column (GE Healthcare). A linear gradient of 2 M to 0 M (NH_4_)_2_SO_4_ in 100 mM phosphate buffer (pH 7.0) was used to resolve trimers from dimers and monomers. The trimer-containing fractions were further purified by SEC as described for His-tagged AD8 SOSIP gp140.

### 2.5. Production of VLPs and Membrane-Bound Env Expression

VLPs were produced in HEK 293T cells with single VLP expression plasmids or cotransfection of an Env-deficient VLP vector and Env-expression plasmid (9:1 DNA mass ratio) and cultured for 48–72 h before particles were concentrated by ultracentrifugation using the reagents and protocols described previously [57]. For experiments in which membrane bound Env expression was validated in the absence of particle expression, HEK 293T cells were transfected as described above with Env expression plasmids alone and cultured for 48 h before harvest. VLPs were produced in Expi293F cells by transfecting with single VLP expression plasmids and cultured for 72 h before being concentrated. A modified Expi293F transfection protocol for VLP production was also used for preparation of VLP immunogens for vaccination: the VLP expression plasmid was cotransfected with an empty plasmid vector (recircularized empty pCRII-TOPO (Thermo Fisher Scientific)) at a mass ratio of 1:15 (VLP plasmid:empty pCRII). This was used to reduce the average copy number of VLP plasmids transfected into each cell to limit the production of contaminating particles.

### 2.6. Western Blotting

For analysis of cell lysate, adherent cells were detached from the surface of culture flasks, washed with ice-cold PBS, and then resuspended in Tris-buffered saline (pH 8.0) supplemented with 0.5% (*v*/*v*) Triton X-100, 5 mM EDTA, and protease inhibitor cocktail (Roche, Basel, Switzerland). Clarified cell lysate or VLP samples were transferred to PVDF membranes (PerkinElmer, Waltham, MA, USA) after SDS-PAGE under reducing conditions using Tris-Glycine-buffered linear, gradient gels according to methods previously detailed [57]. Western blotting was then performed using the indicated antibodies as described previously [57].

### 2.7. Immunization Studies

Mouse immunization studies were approved by The University of Melbourne Animal Ethics Committee (application number 1312863). Groups of 10 female 6–8-week-old BALB/c mice (5 mice per group used for adjuvant only and SOSIP gp140 groups) were obtained from the Animal Resource Centre (Murdoch, Australia) and housed under specific pathogen-free conditions at the BioResources Facility, Department of Microbiology and Immunology, University of Melbourne. The vaccination dose at each timepoint was split between intraperitoneal and subcutaneous routes with a 100 μL volume injected at each site. Equivalent doses of Env were used for SOSIP gp140, and Env-containing iVLP and mVLP immunogens, as determined by anti-gp120 Western blotting. The dose of mVLP Δenv was equalized to the mVLP SOSIP particle number, as determined by p24 ELISA. The dose of ΔVLP (either SOSIP or SOSIPHA) was volume-equalized to the highest volume used for their equivalent iVLP- or mVLP-associated Env (SOSIP or SOSIPHA). Animals were immunized 3 times with a 5 μg Env-equivalent dose at week 0, 2, and 6 followed by a final 20 μg Env-equivalent dose at week 14. The time between each subsequent immunization was increased 2-fold so that immune cells would be exposed to immunogens for a protracted period of time, which would typically occur with a live attenuated virus. For all vaccine doses, a total of 10 μg of murine B-class cytosine-phosphate-guanosine (CpG) oligodeoxyribonucleotide (ODN) 1826 (Miltenyi Biotec, Bergisch Gladbach, Germany) was added to the VLP or protein immunogen diluted in PBS. The lack of detergent or lipid in CpG adjuvant ensured that the VLPs would not be dissolved when formulated together. Blood samples were collected from the tail vein 2 weeks after each immunization.

### 2.8. Enzyme-Linked Immunosorbent Assays (ELISAs)

ELISAs to quantify p24 in viral particle preparations were performed as described previously [57] using recombinant p24 (encoded by the Gag from isolate BH10) purchased from Aalto Bio Reagents. The method for VLP ELISAs was identical to that used previously [57]. Env-equalization was determined by anti-gp120 Western blotting, whereas particle-equalization was determined by p24 ELISA. Bald VLPs (VLPs expressed without Env) were particle-equalized and included to determine nonspecific antibody binding. Env only or ΔVLP preparations were loaded in a volume-equalized manner.

Env-specific antibody titers in mouse sera were determined by indirect D7324 capture ELISA as previously described [72] using either D7324-tagged AD8 SOSIP gp140 or AD8 UNC gp140 as the target antigen. The endpoint dilution titer cut-off was defined as 2.5 times the average OD measured for the same serum dilution of adjuvant-only-vaccination sera sampled at the same time point. The presence of AD8 SOSIP gp140 Env-specific murine IgG1, IgG2a, IgG2b, IgG3, IgA, and IgM were measured in pooled serum from vaccinated mice by indirect D7324 capture ELISA, except the HRP-conjugated goat anti-mouse IgG/A/M (H/L) secondary antibody was substituted with HRP-conjugated isotype-specific secondary antibodies (SouthernBiotech, Birmingham, AL, USA). Significance was determined by a Kruskal–Wallis test followed by Dunn’s multiple comparison test using GraphPad Prism (v7.03) software. Multiple comparisons were performed as follows: ΔVLP SOSIP vs. iVLP SOSIP, ΔVLP SOSIP vs. mVLP SOSIP, iVLP SOSIP vs. mVLP SOSIP, ΔVLP SOSIPHA vs. mVLP SOSIPHA, and mVLP SOSIP vs. mVLP SOSIPHA.

MPER-specific antibody responses in mouse sera were determined by directly coating 200 ng/well MPER peptide (NWFDITNWLWYIK) onto ELISA plates overnight at 4 °C. Plates were blocked before loading serially diluted mouse sera for 2 h followed by anti-mouse antibody detection. The endpoint dilution titer cut-off was defined as 3 times the average OD measured for the same dilution of adjuvant-only-vaccination sera at the same time point.

The avidity of immune serum was estimated by ELISA using a method adapted from previously published protocols [82] and equivalent to the D7324 capture ELISA loaded with D7324-tagged AD8 SOSIP gp140, except that BSA was used instead of skim milk in buffers. Pooled sera from vaccinated mice diluted to contain approximately equal concentrations of Env-specific antibodies were added and incubated for 2.5 h. A range of concentrations (0–3 M) of sodium thiocyanate (NaSCN) diluted in PBS were added to the wells and incubated for 15 min prior to removal by washing and detection of bound mouse serum antibodies. Mouse antibody binding was calculated as a percentage relative to the same sample not exposed to NaSCN.

The serum prevalence of AE clade p24-specific IgG was measured by an ELISA identical to that used to measure p24 in viral particle preparations except for the following modifications: all D7320-coated wells were loaded with solubilized Env-deficient, mature VLPs (mVLP Δenv) diluted to 100 ng/mL p24 and serially diluted vaccinal sera were used instead of the BC1071 primary antibody. The endpoint dilution titer cut-off was defined as 3 times the average OD measured for the same dilution of the adjuvant-only-vaccination group mice sera at the same timepoint. Titers were obtained and statistical tests were performed as described above.

The serum prevalence of antibodies targeting human lipids or proteins presented on the surface of VLPs was measured by VLP ELISA using mVLP Δenv particles as the target antigen coated on the plate. Env-specific mAbs were substituted with sera from vaccinated mice serially diluted in block buffer, and the secondary antibody was substituted with HRP-conjugated goat anti-mouse IgG/A/M (H/L). The endpoint dilution titer cut-off was defined as 3 times the average OD measured for the same dilution of adjuvant-only-vaccination sera at the same time point.

### 2.9. Virus Fusion Assay

Viral particles used in fusion assays were produced by cotransfecting pDRNL4-3ΔenvR-, pMM310 (obtained from NIH HRP) [83], and an Env-expression plasmid at a DNA mass ratio of 9:1:2, respectively. Particles were expressed in 293T cells and concentrated from culture supernatant as described above for VLPs. The viral fusion assays (originally based on published protocols [83]) were performed as described previously [57].

### 2.10. Neutralization Assays

Pseudoviruses (PV) were produced by cotransfection of 293T cells with a 9:1 DNA mass ratio of pDRNL4-3Δenv to an Env expression vector encoding the gp160 of the strain of interest: MN.3 (pSVIII-MN; provided by J. Sodroski, Division of AIDS, Harvard Medical School), SF162.LS (pCAGGS-SF162; provided by L. Stamatatos, Fred Hutchinson Cancer Research Center and C. Cheng-Mayer, Aaron Diamond AIDS Research Center) and AD8 (pCMV-AD8), subsequently referred to in the text as MN, SF162, and AD8, respectively. Virus was also pseudotyped with the amphotropic MuLV envelope glycoprotein to control for nonspecific HIV-1 neutralization by cotransfecting with pSV-A-MLV-*env* (obtained from NIH HRP) [84]. The same transfection protocol used for VLPs was used here, except after the transfection mixture was removed, cells were overlayed with Opti-MEM (Thermo Fisher Scientific) containing 1 × ITS Liquid Media Supplement. At 48 h post-transfection, the PV-containing supernatant was harvested and clarified by centrifugation at 1200× *g* for 10 min at 4 °C, FBS (Thermo Fisher Scientific) was added to a final concentration of 20% (*v*/*v*), and the supernatant was further clarified by passage through a 0.45 μm filter. Aliquots of the clarified PV-containing supernatant were stored at −80 °C until use.

The neutralizing activity of serum from vaccinated mice was assessed using the standardized TZM-bl neutralization assay according to published protocols [85]. Briefly, in flat-bottom 96-well plates, PV (diluted to achieve approximately 150,000 relative luminescence units (RLU)) were incubated for 90 min with serially diluted (starting from 1:25), heat-inactivated (56 °C for 15 min) pooled immune sera from vaccinated mice. For each vaccination group, an equivalent volume of PV was incubated with an identical dilution of pooled, heat-inactivated sera collected before vaccination (preimmune). Each test condition was assayed in duplicate wells. Target TZM-bl cells (1 × 10^4^ cells/well) in media containing DEAE-Dextran (at a final concentration of 12.5 μg/mL when added to the PV–serum mixture) were added to the PV–serum mixtures and cultured for 48–72 h at 37 °C, 5% (*v*/*v*) CO_2_. Luciferase expression from the integrated LTR reporter in TZM-bl cells was measured as RLU using the britelite plus Reporter Gene Assay System (PerkinElmer, MA, USA) and a FLUOstar Omega Microplate Reader (BMG LABTECH GmbH, Ortenberg, Germany). The RLU values were used to calculate percentage neutralization as equal to 1—((immune serum—cell only control)/(preimmune serum—cell only control)), and the dilution of serum required to neutralize 50% of viral infectivity (ID50) was determined by interpolation from fitted sigmoid curves (GraphPad Prism v7.03).

## 3. Results

### 3.1. Envs with Mutated or Chimeric TM and CT Domains Have Increased Incorporation into mVLPs

To examine the ability of alterations to the HIV-1 Env TM and CT domain to influence Env incorporation into mVLPs, the AD8 multicistronic Env/Vpu/Rev expression plasmid (pCMV-AD8-160) was variably modified compared to the wild-type sequence (WT, schematic in Figure 1A). The modifications included removal of CT motifs associated with Env endocytosis [86] (Δendo) or complete truncation of the Env CT (ΔCT) (shown to enhance Env incorporation into virions [87]), as well as substitution of the HIV-1 TM/CT with sequences from MMTV Env and IFA HA (shown to enhance the incorporation of uncleaved HIV-1 Env into Baculovirus-expressed HIV-1 VLPs [33]) (MMTV-TMCT and HA-TMCT, respectively). Finally, the AD8 Env TM and CT were replaced with the 93TH253.3 strain *env* TM/CT sequences, given the mVLP Gag was derived from this strain (AE-TMCT). The expression of the WT and modified Envs from transfected 293T cells was analysed by Western blotting on cell lysates (Figure 1B). All the modified Env vectors expressed Env, including the HA-TMCT and MMTV-TMCT vectors, indicating the *rev* splicing and mRNA expression were not negatively impacted. The amount of gp120 expression was similar between WT and modified Env sequences except for a noticeable increase in gp120 expressed for HA-TMCT Env and a lesser increase for Δendo Env. The amount of uncleaved Env (gp160) was similar between the WT, Δendo, MMTV-TMCT, and AE-TMCT Env vectors, with gp120-gp41 cleavage efficiencies ranging between 53 and 66%. The ΔCT and HA-TMCT Envs showed a decreased amount of gp160 and enhanced cleavage efficiencies of 81% and 85%, respectively. As expected, the apparent size of the uncleaved Env differed depending on the specific CT modification: deletion of the CT (ΔCT) or replacement with the shorter HA CT (HA-TMCT) showed an ~10kDa size decrease, whereas the MMTV-TMCT had less affect, as the MMTV CT was longer than that of HA. The predominant band under the HA-TMCT gp120, and to a lesser extent under the MMTV-TMCT gp120, was potentially due to partial proteolytic clipping of gp120 that often occurs in the V3 region of subtype B Env proteins [88,89,90]. Mock-transfected cells did not show any gp120 or gp160 staining, and the relative intensity of GAPDH was similar between all samples.

To assess the ability of the modified Envs to be incorporated into mVLPs, the Env plasmids were cotransfected with the Env-deficient mVLP (mVLP Δenv) expression plasmid into 293T cells. Anti-gp120 and anti-gp41 Western blotting of mVLPs (equalized by p24 ELISA) was used to visualize VLP-associated Env (Figure 1C). The gp160 (uncleaved), gp120, and gp41 Env species on these blots were analyzed by densitometry to determine the relative change in incorporation of each Env species (Figure 1D). As noted above, the band observed below the gp120 was possibly the result of partial V3 clipping and was not included in the analysis. Surprisingly, the ΔCT modification, which is commonly used to increase Env surface expression and virion incorporation [87,91,92], showed approximately one-fifth the incorporation of gp120 and gp160, but similar gp41 incorporation relative to WT. The Δendo and MMTV-TMCT Envs were more effective at increasing the gp41 incorporation, with 1.9- and 3.5-fold increases, respectively; however, they failed to increase gp120 incorporation above WT levels. MMTV-TMCT Env showed a similar reduction in gp120 incorporation (0.2-fold of WT) to that seen for ΔCT Env. The HA-TMCT Env showed increased gp41 (3-fold) and gp120 (2-fold) incorporation and a slightly higher cleavage efficiency of Env, given there was no increase in gp160 staining relative to WT. The AE-TMCT Env did not show any enhancement of Env incorporation into the mVLPs despite the Gag polyprotein expressed by the VLPs being derived from the same strain as the TM and CT. The general trend observed for Env TM and CT modifications facilitating improvements in gp41 incorporation was that there was a lesser corresponding increase in gp120 loading. This suggested that while gp41 retention on the cell (and subsequently VLP) surface was increased, the continued occurrence of gp120 shedding led to a population of gp41 stumps that with WT TM/CT domains would normally be endocytosed before virion incorporation.

### 3.2. Introducing Stabilizing Env Mutations to Anchor gp120 to VLP Membrane Improves Env Retention

To address the presumed gp120 shedding, two different gp120-gp41 stabilizing mutations were employed to anchor gp120 to the gp41 domain (Figure 2A): introduction of a disulfide bond between the gp120 and gp41 domains through introduction of A501C and T605C amino-acid substitutions (SOS) [80], or mutation of the cellular protease cleavage site between the gp120 and gp41 domains via K501T and R511G amino-acid substitutions (UNC) [93]. The SOS and UNC modifications were assessed on the WT and modified Envs above, apart from AE-TMCT Env, given its overall low incorporation into VLPs (Figure 1D). The incorporation of SOS and UNC Env into mVLPs was assessed by Western blotting (Figure 2B,C, respectively) as described in the previous section, with only gp160 bands for UNC Envs analysed by densitometry, given that cleavage into gp120 and gp41 subunits was eliminated. When coupled with the SOS mutation, the Δendo and ΔCT modifications showed, on average, similar improvements in gp41 incorporation, with 2.6- and 2.8-fold increases relative to WT, respectively (Figure 2D). The Δendo demonstrated modest increases in gp160 and gp120 incorporation (1.6- and 1.4-fold, respectively), whereas ΔCT showed no increase in gp160 or gp120 incorporation compared to WT. Both the SOS-stabilized MMTV-TMCT and HA-TMCT Env achieved large increases in gp41 incorporation (11- and 13-fold, respectively), but lacked comparable improvements in gp120 incorporation (1.1- and 1.8-fold, respectively). Both chimeric TM/CT modifications also showed increased gp160 incorporation (3- and 5.7-fold, respectively) relative to WT, and when further compared to each of their gp120 incorporation results, showed that the gp120-gp41 cleavage efficiency was reduced by these modifications in combination with the SOS mutation. Envs with the UNC mutation showed sizable increases in Env incorporation for each of the TM/CT modifications tested (Figure 2E). Of the two modifications designed to abolish HIV-1 Env CT endocytosis functions, the ΔCT Env showed a 23-fold increase in Env incorporation relative to WT, whereas the Δendo Env only showed a 13-fold increase. The HA-TMCT Env showed the highest increase in Env incorporation (33-fold compared to WT), whereas the MMTV-TMCT Env demonstrated a similar level of incorporation as ΔCT Env.

### 3.3. Cleaved and Uncleaved VLP-Associated Env Antigenicity Is Enhanced with Introduction of the SOSIP Mutation

**Figure 3:**
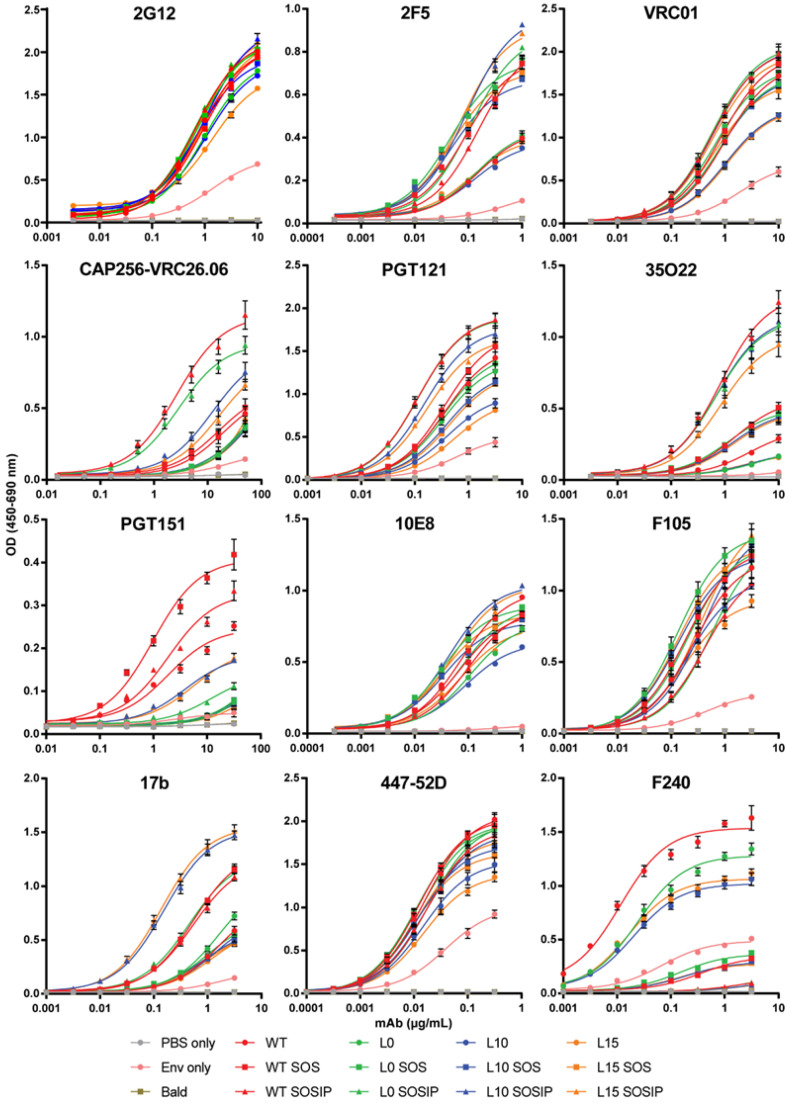
Comparison of neutralizing and non-neutralizing antibody binding to mVLP-associated linker-stabilized Env. VLP ELISA binding curves of bNAbs (2G12, 2F5, VRC01, CAP256-VRC26.06, PGT121, 35O22, PGT151, 10E8) and non-neutralizing mAbs (F105, 17b, 447-52D, F240) to a buffer-only control (PBS only), mVLPΔenv that does not present Env on its surface (Bald), mVLPΔenv pseudotyped with linker-stabilized and cleavage-competent gp160 vectors detailed in Figure 2A, and a wild-type AD8 Env vector only preparation controlling for microvesicles and exosomes containing Env (Env only). ELISAs were performed using 100 ng/well of gp120 as determined by anti-gp120 Western blotting. Bald sample loading was equalized to the highest p24 loading as determined by p24 ELISA, and loading of the Env-only sample was volume-equalized to WT (which contains the same gp160 sequence). Data represent the mean and SEM of 3 independent experiments.

The substantial increase of UNC-stabilized Env incorporation into mVLPs with the HA-TMCT modification prompted us to seek further modifications that could improve the antigenicity of the VLP-associated Env, given that uncleaved Env is not representative of the functional, cleaved Env that bNAbs often target [9]. Work from other groups has demonstrated for soluble Env that the antigenicity of cleaved, native-like trimers could be approximated by introducing flexible amino-acid linkers between uncleaved gp120 and gp41 ectodomains, as well as SOS and I559P (SOSIP) mutations [94]. Membrane-bound Env expression vectors with flexible linker sequences of either 10 (L10), 15 (L15), or no (L0) amino acids between the gp120 and gp41 domains were generated in the presence and absence of the SOSIP mutation or the SOS mutation alone. As controls, cleavage-competent Env vectors without any modification (WT), with a SOS mutation (WT SOS), or a SOSIP mutation (WT SOSIP) were included (Env schematics are summarized in Figure S1A). The antigenicity of the linker Envs was assessed by cotransfecting the Env expression vectors with the mVLP Δenv vector into 293T cells and performing Env-equalized VLP ELISAs with a panel of neutralizing and non-neutralizing mAbs (curves shown in Figure 3 and binding summarized in Table 1 as fold-change compared to WT). To control for the presence of microvesicles and exosomes bearing Env, an Env-only (WT Env plasmid, VLP expression vector substituted for an empty vector) preparation was included. The 2G12 binding relative to WT confirmed the VLPs were relatively well Env-equalized (given the carbohydrate-only epitope of 2G12 is less dependent on Env conformation [95]), and the Env-only binding curve indicated the vast majority of the ELISA reactivity was mediated by VLP-associated Env rather than Env presented on cellular exosomes or microvesicles (Table 1). Binding to 2F5 was enhanced approximately 2-fold for Envs bearing either an SOS or SOSIP mutation, which was consistent with previous observations of the SOS mutation increasing the exposure of the gp41 MPER [96]. The 10E8 binding, also targeting the MPER, was relatively similar across Envs except for L0, L10, and L15, which only showed 0.75-fold of the WT signal or less. VRC01 and PGT121 binding were consistent between the Envs apart from L10 and L15, which were ~70% of the WT value for VRC01 and ~60% of the WT amount for PGT121. The binding of gp120-gp41 interface antibody 35O22 also showed decreased binding of ~60% for L10 and L15 along with L0, whereas any Env bearing the SOS showed improved binding approaching 2-fold compared to WT and SOSIP Envs were enhanced between 3.7- and 4.8-fold. The WT SOSIP and L0 SOSIP were the best binders at 4.84- and 4.38-fold of WT, respectively. These two Envs additionally showed the greatest improved binding (3.62- and 2.96-fold of WT, respectively) to the bNAb CAP256-VRC26.06 (subsequently referred to as VRC26.06), which recognizes a trimeric quaternary V1V2 epitope [69] and so is indicative of compact, native-like trimer conformation. The WT SOS, L10 SOSIP, and L15 SOSIP also showed increased VRC26.06 binding, albeit less than 2-fold of WT. Conversely the L0, L10, or L15 with or without SOS displayed reduced VRC26.06 activity. The binding of non-neutralising antibodies to the Env mutants showed relatively similar efficiency of F105 and 447-52D binding (all within ~0.7 and 1.3-fold of the WT Env). There was around 2-fold increased binding of 17b by WT SOS, WT SOSIP, and L0 SOSIP, while more than 3-fold enhanced 17b binding was observed for L10 SOSIP and L15 SOSIP. The binding of F240 was similar to WT with L0 Env, and mildly reduced for L10 and L15 Envs. The SOS mutation in any Env was effective at reducing F240 binding to ~20% of WT, while all Envs with the SOSIP mutation showed only ~4% of the WT binding signal to F240.

Area under the curve (AUC) was calculated from mean ELISA binding curves (Figure S2) and used to determine fold-change. Fold-change was calculated relative to WT Env AUC. Fold-reductions in binding are shaded green and fold-increases are shaded red (intensity proportional to change). Neutralizing antibodies are shown in the top rows of the table and non-neutralizing antibodies are below, separated by a dashed line.

Given the VLP-associated L10 and L15 SOSIP Envs (AD8 strain) did not show superior trimer-specific (VRC26.06) antibody binding compared to the shorter-linker L0 SOSIP Env in contrast to reports for soluble (BG505 strain) Env proteins [94], the linker and SOS/SOSIP modifications were assessed in a soluble-protein setting to determine whether this difference was due to genotype differences or presentation on the VLP membrane versus soluble Env. The linker Env mutants (Figure S1A) were expressed as D7324-tagged soluble gp140s, and unpurified supernatants were antigenically screened by D7324 capture ELISA. The similar VRC01 binding curves observed for all gp140 proteins (Figure S1B) demonstrated that similar amounts of soluble Env were captured from the supernatant, given the VRC01 epitope is similarly displayed on monomers or higher-order oligomers [70] and between gp140 proteins with different linker lengths [94]. When binding was assessed using the bNAb VRC26.06, the WT SOSIP demonstrated the highest binding of the tested gp140 proteins, which was consistent with its performance in VLP ELISAs when expressed on mVLPs (Table 1 and Figure 3). In contrast, the L0 SOSIP gp140 showed no reactivity with VRC26.06, and the L10 and L15 gp140 proteins with the SOSIP mutation exhibited low binding (Figure S1B). All the other gp140 proteins displayed no greater VRC26.06 activity than the Mock supernatant. This suggested the unexpected ability of uncleaved and shorter linker-stabilized VLP-associated AD8 strain Env to react with trimer-specific mAbs was likely due to differences in Env conformation when presented on the VLP membrane. The SOSIP-modified Envs, which bound strongly to most bNAbs (Table 1), generally showed reduced or unchanged binding to non-neutralizing mAbs, except for 17b binding. Despite the initial intention of using VLP-presented uncleaved Env sequences, given their superior incorporation when containing the HA-TMCT modification, the overall increased bNAb epitope exposure exhibited by the cleavage-competent SOSIP-containing Env (WT SOSIP) led us to select this Env for use in further experiments.

### 3.4. Env Antigenicity but Not Function Is Preserved When Combining SOS and SOSIP Stabilization with HA TM and CT Domain

**Figure 4:**
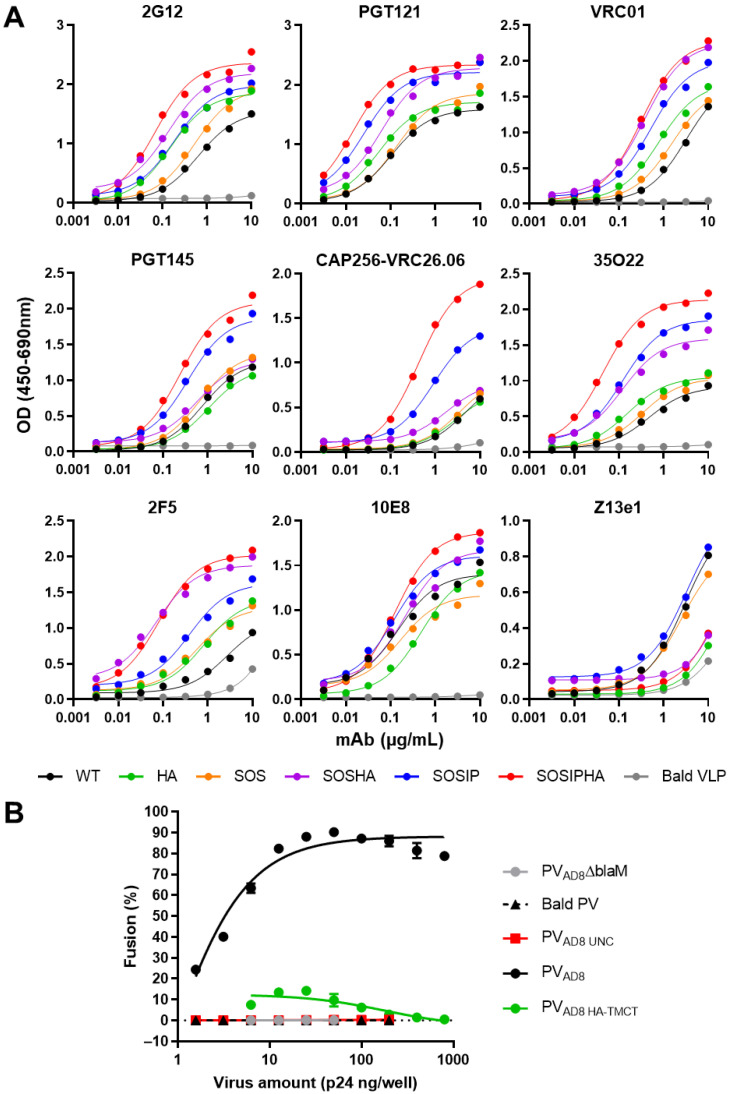
Antigenicity and fusion function of Env with or without a HA TM and CT domain expressed on mVLPs and HIV-1 pseudovirions. (**A**) Representative ELISA binding curves of VRC01, PGT121, 2G12, PGT145, CAP256-VRC26.06, 35O22, 2F5, 10E8, and Z13e1 to mVLP particles expressing cleavage-competent gp160 without (WT) or with a SOS or SOSIP modification (SOS or SOSIP, respectively), or the same set of gp160 sequences containing a haemagglutinin (HA) TM and CT (HA, SOSHA, and SOSIPHA, respectively), and mVLPΔenv particles (Bald VLP). VLP ELISAs were performed using an equal number of particles/well as determined by p24 ELISA (data not shown). (**B**) Fluorescent fusion assays with NL4-3 pseudovirus bearing no Env (Bald PV), uncleaved AD8 gp160 (PV_AD8_ UNC), wild-type AD8 gp160 (PV_AD8_), and wild-type AD8 gp160 containing a HA TM and CT (PV_AD8 HA-TMCT_). PV_AD8_ΔblaM particles lacked the BlaM-Vpr fusion protein. The amount of virus was determined by p24 ELISA, and CEM.NKR CCR5+ cells were used as target cells. Values represent the mean and SEM of duplicate measurements.

In order to assess whether the antigenic properties of Env were maintained when VLP incorporation was enhanced with the HA-TMCT modification, combinations of WT, SOS, and SOSIP Env with or without the HA-TMCT modification (HA, SOSHA, SOSIPHA or WT, SOS, SOSIP, respectively) were expressed on mVLPs by cotransfection with the mVLP Δenv vector into 293T cells. The antigenicity was measured via VLP ELISAs with a panel of bNAbs on the VLPs as described earlier. The binding curves for 2G12 showed that increasing amounts of Env were presented on the mVLP surface with the introduction of the SOS and SOSIP mutations, respectively (Figure 4A). Consistent with previous experiments that showed increased Env incorporation with the HA-TMCT modification, the 2G12 binding was enhanced for each Env form (WT, SOS, and SOSIP) that contained the HA-TMCT. A similar distribution of binding curves for the Envs was observed for VRC01 and 2F5, suggesting their epitopes were equally displayed. The spread of binding curves for PGT121 and 35O22 were also similar to that of 2G12, with the exception that the SOSIP Env showed ~2-fold higher binding than the SOSHA Env, and the SOS Env exhibited a smaller increase in binding over the WT Env (compared to 2G12). The SOSIP and SOSIPHA Envs demonstrated the highest binding to the quaternary epitope bNAbs PGT145 and VRC26.06, with SOSIPHA showing the highest binding, given the greater level of Env displayed on the mVLP surface. All other Envs showed similarly low binding to these bNAbs. Analysis with the gp41 MPER-specific bNAbs 10E8 and Z13e1 yielded differing binding efficiencies compared to those for gp120-specific bNAbs. The binding of Z13e1 was relatively similar whether the Env contained a SOS/SOSIP mutation and was very weak overall; however, binding was completely abolished when the HA-TMCT modification was present. Except for Z13e1, the overall mAb panel showed the HA-TMCT modification in combination with the SOSIP mutation did not appear to negatively impact the presentation of gp120 and gp41 bNAb epitopes.

The effect of the HA-TMCT modification on Env function was assessed using a fluorescent fusion assay with CEM.NKR CCR5+ cells as the target cells. The SOS and SOSIP mutations were not investigated, since previous reports have shown the SOS mutation inhibits virion fusion unless Env is exposed to a reducing agent after CD4 engagement [97,98]. Similarly, the I559P mutation has been reported to be nonfunctional [99]. The WT and HA Env expression plasmids were cotransfected in 293T cells with an Env-deficient NL4-3 virion and BlaM-Vpr fusion protein-expression vectors to produce PV_AD8_ and PV_AD8 HA-TMCT_ pseudovirus, respectively. Particles pseudotyped with uncleaved Env (PV_AD8 UNC_) and an empty vector (Bald PV) were used as negative controls for fusion. Pseudovirus bearing a functional Env but no BlaM-Vpr fusion protein were also generated (PV_AD8_ΔBlaM). As expected, the Bald PV and PV_AD8 UNC_ showed no fusion ability, given the lack of Env or presence of uncleaved Env (which is nonfunctional), respectively (Figure 4B). Pseudovirus expressing WT Env were able to mediate fusion efficiently over the range of p24 amounts assayed (and saturated the assay with less than 100 ng p24/well being used), whereas the same amounts of pseudovirus bearing the HA-TMCT-modified Env exhibited vastly reduced fusion activity that did not exceed 15% fusion. While the fusion activity of HIV-1 Env with heterologous TM domains has not been reported to date, this result is consistent with observations that interaction between the HIV-1 Env fusion peptide and TM domain is important in mediating virus–cell membrane fusion [100].

### 3.5. Suspension Cell Expression of VLPs Preserve bNAb Recognition and Can Reduce Contaminating Non-VLP Particles

Single plasmid mVLPs were produced in 293T cells with the modified Envs (SOS, SOSIP, HA, SOSHA, and SOSIPHA) cloned into the VLP vectors. At the same time, an “Env-only” preparation was made for each Env by expressing an Env-matched VLP expression construct lacking Gag expression (ΔVLP) (schematic with WT Env shown in Figure S3A). VLP ELISAs were performed using p24-equalized amounts of the mVLPs. Antigenicity was measured with VRC01 mAb, as the VRC01 epitope is similarly displayed on monomers and higher-order oligomers [70], such that any potential differences in the proportion of Env trimers between VLP- or extracellular-vesicle-associated Env should not affect the comparison of their Env levels. The single-plasmid-expressed mVLPs all produced Env efficiently whether encoding a wild-type TM and CT (Figure S3B) or the HA-TMCT modification (Figure S3C). Additionally, for both TM/CT sequences, the SOS and SOSIP mutations demonstrated increased VRC01 binding relative to Env without SOS/SOSIP modifications, suggesting higher Env levels were present. However, the ΔVLP showed much higher reactivity to VRC01 than was previously observed with Env-only preparations (Table 1), suggesting similar levels of Env were present in mVLP/ΔVLP preparations regardless of the Env expressed. The antigenicity of WT and SOSIP Env presented on the presumed contaminating microvesicles and exosomes was further assessed with other bNAbs targeting epitopes distinct from the CD4bs. To ensure the similar Env particle-expression level between mVLP and ΔVLP preparations was not simply due to relatively low production of Env from the mVLP expression cassette, the VLP ELISAs also measured Env presentation on immature VLPs (iVLPs). The 2G12 and VRC01 binding were comparable for WT Env whether displayed on mVLPs, iVLPs, or ΔVLPs (Figure S4A). Conversely, the ΔVLP WT Env showed a marked reduction in 35O22 binding, while Env presented on iVLP and mVLP bound the mAb similarly. The ΔVLP WT (and iVLP WT) also bound PGT145 less efficiently than mVLP WT, suggesting the proportion of trimeric Env differed across the particles. A similar but more exaggerated trend was observed with SOSIP Env: 2G12 and VRC01 reactivity was consistent between mVLP, iVLP, and ΔVLP samples, whereas the ΔVLP SOSIP showed reduced binding to PGT145 and 35O22 as compared to either mVLP SOSIP or iVLP SOSIP (which showed very similar binding to each other) (Figure S4A). Overall, the relative antigenicity of ΔVLP Env compared to mVLP or iVLP Env suggested the amount of microvesicle- and exosome-associated Env expressed from 293T cells transfected with a single plasmid was greater than when cotransfections were performed, but the conformation of Env on these contaminants was somewhat distinct to HIV-1 viral-particle-associated Env.

The use of suspension cell line expression systems typically yields higher concentrations of secreted products in the culture supernatant than do adherent cell lines, therefore the expression of VLPs from a high-density suspension cell line (Expi293F) was investigated as a potential method to increase the proportion of HIV-1 VLPs relative to microvesicle- and exosome-associated Env contaminants when expressing VLPs from a single vector. The amount of contaminating-particle-associated Env produced by Expi293F cells was assessed using the same VLP ELISA approach described above with mVLPs, iVLPs, and ΔVLPs. Mirroring the results seen for 293T-produced particles (Figure S4A), the binding for VRC01 and 2G12 was similar between iVLP, mVLP, and ΔVLP preparations, although the SOSIP Env-bearing particles showed higher reactivity per particle (Figure S4B). The PGT145 and 35O22 binding was higher to particles expressing SOSIP Env and followed the same trend seen for 293T-produced particles with mVLP and iVLP binding being similar to each other, whereas the ΔVLPs showed reduced reactivity. This showed that the higher yield of particles from Expi293F cells was also accompanied by a similarly increased yield of contaminating particles. The quantity of Env presented on the Expi293F-produced VLPs was then assessed to ensure the suspension-cell-derived VLPs incorporated similar amounts of Env by comparing equal numbers of mVLP SOSIP and mVLP SOSHA particles produced in 293T or Expi293F cells. VLP ELISAs using bNAbs VRC01, VRC26.06, and 10E8 confirmed the VLPs presented similar levels of Env, as demonstrated by equivalent mAb binding curves for mVLPs bearing identically modified Env (SOSIP or SOSHA) from either cell line (Figure S4C).

We next devised a method to reduce contaminating Env-bearing particles from VLP preparations based on the initial observation that the contaminants were present at higher levels when single-plasmid expression vectors were used as compared to cotransfecting with Env-deficient VLP and Env expression plasmids. In the cotransfection setting, the amount of Env expression plasmid was typically one-tenth the quantity of the VLP expression plasmid, meaning single-plasmid transfections represented a significant increase in the copy number per cell of Env DNA. Reduced plasmid copy number transfections were performed with equal numbers of Expi293F cells by 2-fold serial dilution (from 100% to 3.125%) of mVLP SOSIP or ΔVLP SOSIP expression plasmids with an empty vector plasmid that did not contain any mammalian promoters. The Expi293F cells were used here since they had previously demonstrated greater yields of VLPs per mL of supernatant compared to 293T cells, which was expected to partially offset the reduction in VLP yield as the plasmid copy number was reduced. As expected, the yield of mVLPs was proportional to the amount of VLP expression plasmid transfected with decreasing p24 concentration in the concentrated mVLP SOSIP preparations decreasing more than 10-fold as the proportion of VLP expression plasmid decreased from 100% to 3.125% of total DNA (Figure S5A). VRC01 VLP ELISA was used to compare the reactivity of the mVLP SOSIP and ΔVLP SOSIP preparations. The reactivity of mVLPs to VRC01 was proportionally lower as VLP plasmid copy number was reduced (Figure S5B), which was consistent with the lower p24 concentrations. However, there was a progressive increase in binding of the mVLPs compared to ΔVLPs as the proportion of VLP plasmid transfected decreased. In particular, the 6.25% mVLP/ΔVLP pair showed negligible ΔVLP binding while still retaining an acceptable yield of mVLP and VRC01 reactivity. Therefore, these Expi293F plasmid transfection conditions were utilized for subsequent expression of VLPs.

### 3.6. Neutralizing Antibody Epitope Exposure Is Preserved on SOSIP-Stabilized Env When Expressed on iVLPs and mVLPs with HA TM and CT

**Figure 5:**
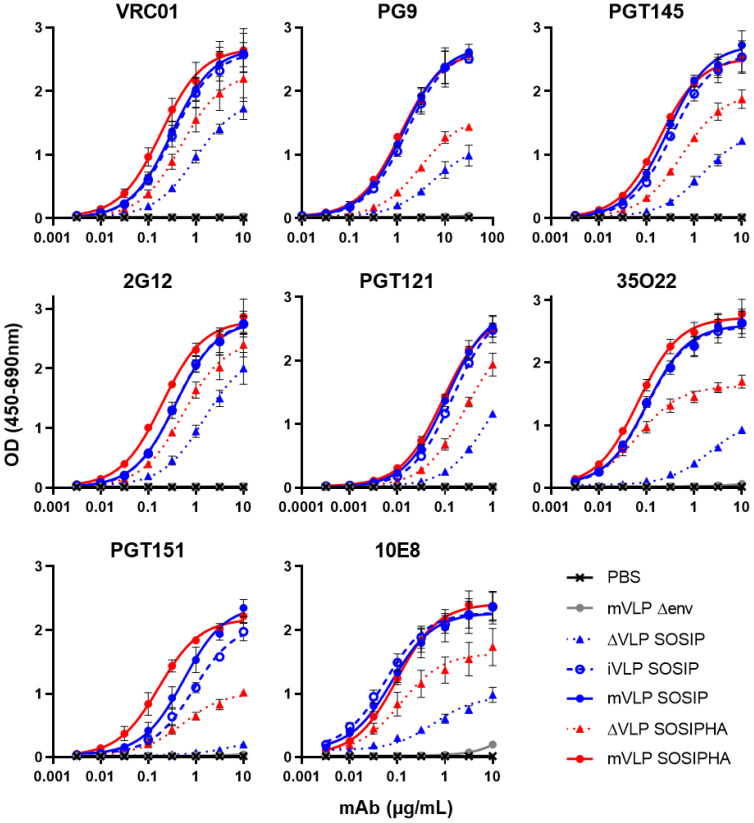
Antigenicity of single plasmid-expressed VLP immunogens: VLP ELISA binding curves of VRC01, PG9, PGT145, 2G12, PGT121, 35O22, PGT151, and 10E8 to Expi293F-produced ΔVLPs and mVLPs bearing SOSIP or SOSIPHA Env, and iVLPs bearing SOSIP Env. A buffer-only control (PBS) and Expi293F-produced mVLPΔenv particles were also assayed. 200 ng equivalent gp120/well (as determined by anti-gp120 Western blotting) was loaded for iVLP SOSIP, mVLP SOSIP and mVLP SOSIPHA. ΔVLP loading was volume-equalized to the highest volume used for their equivalent VLP-associated Env (SOSIP or SOSIPHA). mVLP Δenv loading was p24-equalized to mVLP SOSIP p24 as determined by p24 ELISA. Values represent the mean and SEM of 3 independent experiments.

To compare the exposure of bNAb epitopes on mVLPs expressing SOSIP and SOSIPHA Env and iVLP expressing SOSIP Env, VLP ELISAs were performed. These VLPs (and ΔVLPs expressing SOSIP and SOSIPHA Env) were produced from Expi293F cells using the optimized transfection conditions (determined in the previous section). The binding of gp120-specific bNAbs VRC01, PG9, PGT145, 2G12, and PGT121 were highly similar for iVLP SOSIP, mVLP SOSIP, and mVLP SOSIPHA, indicating that neither the VLP morphology (immature or mature) nor the HA TM/CT substitution substantially influenced the presentation of the epitopes of these antibodies (Figure 5). In contrast, the bNAbs with epitopes overlapping gp120-gp41 diverged from this trend, as demonstrated by enhanced PGT151 binding to mVLP SOSIPHA relative to mVLP SOSIP and reduced binding to iVLP SOSIP compared to mVLP SOSIP, whereas 35O22 binding was similar between all three VLPs. Given the dependence of PGT151 on cleavage between gp120 and gp41 domains, this suggested the HA TM/CT modified Env had a slightly more efficient cleavage compared to SOSIP-only Env, while iVLP-associated SOSIP Env was less efficiently cleaved relative to mVLP-associated SOSIP Env. The results for the gp120-specific bNAbs showed the binding of the gp41-specific 10E8 was very similar between iVLP SOSIP, mVLP SOSIP, and mVLP SOSIPHA. The difference in 2G12 reactivity between iVLP SOSIP or mVLP SOSIP and the ΔVLP SOSIP control preparation was not as large as observed in the smaller-scale copy number titration experiments (Figure S5B, see 6.25%), likely due to differences in the cell growth at larger scale. Nonetheless, it did represent a reduction in contaminating-particle-associated Env compared to VLPs produced prior to optimization of the transfection conditions. Interestingly, the reduced plasmid copy number transfection appeared less effective at reducing ΔVLP SOSIPHA reactivity relative to mVLP SOSIPHA, with a lower reduction in 2G12 binding being observed as compared to the decrease seen for ΔVLP SOSIP relative to mVLP and iVLP SOSIP. A similar difference in antigenicity was observed between iVLPs or mVLPs and their respective ΔVLPs for VRC01 and PGT121, whereas PG9, PGT145, 35O22, PGT151, and 10E8 showed larger differences in their binding to these particles (AUC changes summarized in Table 2). The most extreme examples of these were 35O22 and PGT151, which both exhibited significantly reduced binding to ΔVLP SOSIP despite showing strong binding to both iVLP SOSIP and mVLP SOSIP. This was consistent with earlier observations (prior to optimization of the plasmid transfection conditions) of 293T and Expi293F cell-produced ΔVLPs demonstrating specific reductions in binding to PGT145 and 35O22, while their binding to VRC01 and 2G12 were similar to iVLPs and mVLPs (Figure S4A,B).

### 3.7. Vaccination with VLPs Bearing SOSIP-Stabilized Env Induces Lower Serum Titers of Env-Specific Antibodies Compared to Soluble SOSIP gp140

**Figure 6:**
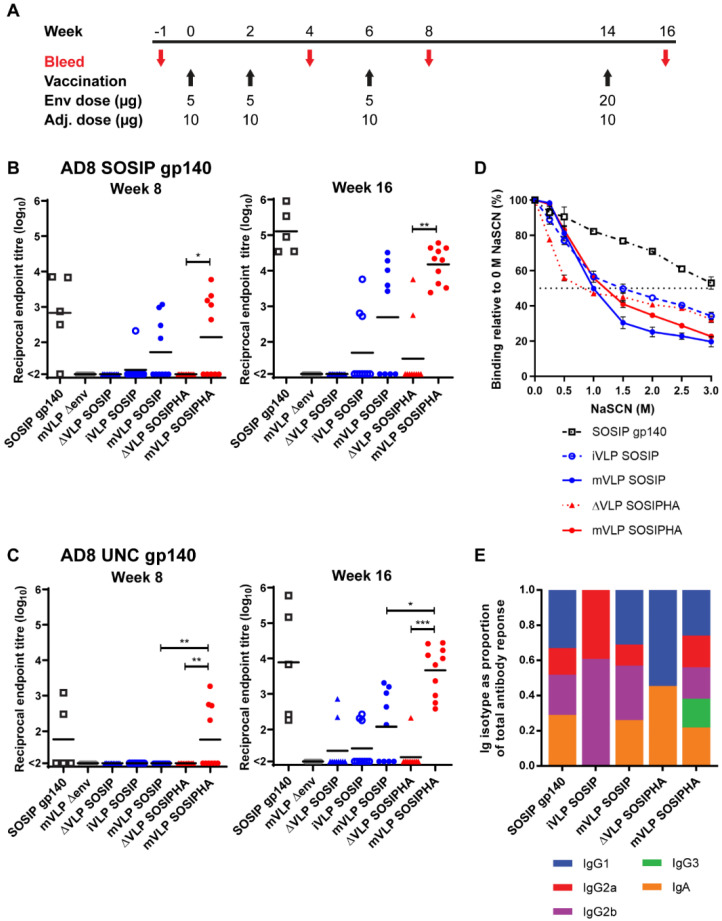
Mouse vaccination schedule and serum Env-specific antibody responses. (**A**) Mice were vaccinated 4 times and blood was collected at the indicated time points. The dose of Env and CpG 1826 ODN adjuvant are indicated below each vaccination (n = 10 per group; except adjuvant-only and SOSIP gp140, *n* = 5). Reciprocal endpoint serum antibody titers (log_10_-transformed) at week 8 and week 16 against (**B**) D7324-tagged AD8 SOSIP gp140 or (**C**) AD8 UNC gp140 as determined by D7324-capture ELISA. Each point represents an individual animal. The black bar represents the mean titer. The endpoint dilution titer cut-off was defined as 2.5 times the average OD measured for the same serum dilution of the adjuvant-only-vaccination group mice sera sampled at the same time point. The lowest reciprocal dilution assayed was 100 and titers less than 100 were recorded as “<2”. Significance was determined by a Kruskal–Wallis test followed by Dunn’s multiple comparison test. *p*-values are indicated as *p* < 0.05 (*), < 0.01 (**), < 0.001 (***). Multiple comparisons were performed as follows: ΔVLP SOSIP vs. iVLP SOSIP, ΔVLP SOSIP vs. mVLP SOSIP, iVLP SOSIP vs. mVLP SOSIP, ΔVLP SOSIPHA vs. mVLP SOSIPHA, and mVLP SOSIP vs. mVLP SOSIPHA. (**D**,**E**) Week 16 sera from animals with AD8 SOSIP gp140 antibody titers ≥2 were pooled within vaccination groups. (**D**) Sera were analysed in an Env-specific NaSCN-displacement ELISA. D7324-tagged AD8 SOSIP gp140 was captured on the plates. Each sample was diluted to give a similar OD in the absence of NaSCN. Values represent the mean, and error bars show the SEM from 2 independent experiments. (**E**) Isotype-specific reciprocal endpoint serum antibody titers at week 16 were determined using D7324-tagged AD8 SOSIP gp140 in a D7324-capture ELISA. The endpoint dilution titer cut-off was defined as 2.5 times the average OD measured for the same serum dilution of the adjuvant-only-vaccination group mice sera sampled at the same time point. The lowest reciprocal dilution assayed was 100. Titers less than 100 were recorded as “0”. The isotype as a proportion of the total antibody response was calculated by dividing each individual antibody isotype titer by the sum of all antibody isotypes for a given vaccination group. For (**B**,**C**,**E**), data are representative of 2 independent experiments.

Mice were vaccinated using iVLP SOSIP, mVLP SOSIP, and mVLP SOSIPHA particles as immunogens to compare the immunogenicity of Env presented on immature and mature VLPs (iVLP SOSIP versus mVLP SOSIP) and of low and high VLP surface Env density (mVLP SOSIP versus mVLP SOSIPHA) (Figure 6A). Control groups for responses to contaminating-particle-associated Env received volume-equalized doses of ΔVLP SOSIP and ΔVLP SOSIPHA preparations, and one group received mVLP Δenv particles (equalized to the p24 content of the mVLP SOSIP dose) to assess the elicitation of non-Env-specific antibody responses (Table 3). CpG 1826 ODN, which is a murine Toll-like receptor 9 (TLR9) agonist, was used as an adjuvant. A group of 5 mice received the adjuvant in isolation to determine the nonspecific humoral responses in antibody titer assays. An additional control group of 5 mice were vaccinated with soluble AD8 SOSIP.664 gp140 (which contained the same SOSIP modification as Env presented on the VLPs) to compare soluble and VLP-associated Env responses when both were adjuvanted with CpG 1826 ODN.

Modest anti-AD8 SOSIP.664 gp140 (subsequently abbreviated to SOSIP gp140) serum antibody titers ranging from 1:128 to 1:6309 were observed at week 8 for 4 mice in the SOSIP gp140 group and for no more than half of the animals in the iVLP SOSIP, mVLP SOSIP, and mVLP SOSIPHA groups (Figure 6B). However, there were no significant differences other than mVLP SOSIPHA versus ΔVLP SOSIPHA, since no responses were above the threshold in the latter group. The responses to uncleaved AD8 gp140 (UNC gp140) were lower at the same time point, with less than half the animals in the SOSIP gp140 and mVLP SOSIPHA vaccination groups showing above-threshold titers (Figure 6C). Given the limited antibody responses observed after 3 immunizations, the final boost protein concentration was increased 4-fold to better induce a response across all the study groups.

At week 16, the SOSIP gp140 titers were higher, with all mice demonstrating above-threshold titers in the SOSIP gp140 and mVLP SOSIPHA groups, with titers ranging between 1:33,884 and 1:912,010, and 1:2398 and 1:60,255, respectively (Figure 6B). The mVLP SOSIPHA titers were significantly higher than those seen in the ΔVLP SOSIPHA group, with only two mice in the latter group having titers above threshold. The iVLP SOSIP and mVLP SOSIP groups showed increased titers and an increase in animals with detectable titers; however, there was no significant difference in the titers between these groups or between the mVLP SOSIP and mVLP SOSIPHA groups. The UNC gp140 responses for SOSIP gp140 and mVLP SOSIPHA at week 16 were similar to their SOSIP gp140-specific responses, though the bottom of the range of titers was lower (Figure 6B). Detectable UNC gp140-specific antibodies were observed for ΔVLP SOSIP-, iVLP SOSIP-, mVLP SOSIP-, and ΔVLP SOSIPHA-vaccinated animals, yet there were no significant differences between the iVLP SOSIP and mVLP SOSIP groups, or between these groups and their ΔVLP SOSIP control group. Nevertheless, the mVLP SOSIPHA anti-UNC gp140 titers were significantly higher than both the mVLP SOSIP and ΔVLP SOSIPHA groups, with only a single animal in the latter group having an above-threshold titer. Though there were differences in the range of antibody responses to SOSIP and UNC gp140 for the SOSIP gp140-, iVLP SOSIP-, mVLP SOSIP- and mVLP SOSIPHA-vaccinated animals, Spearman’s rank-order correlation testing showed these groups all exhibited significant positive correlation between responses to these two antigens (Figure S6). Finally, given the VLP-associated Env immunogens presented the complete gp41 ectodomain, whereas the AD8 SOSIP.664 gp140 immunogen contained a truncation prior to the gp41 MPER, the serum antibody responses against the gp41 MPER were assessed to determine whether there was any specific targeting of this bNAb epitope for VLP-vaccinated animals. Using the serum collected at week 16, no MPER peptide-specific responses were detected above the assay threshold for any of the vaccination groups, suggesting the inclusion of the MPER epitope on VLP immunogens did not appear to induce anti-Env antibodies targeting this site.

### 3.8. Env Presented on mVLPs Induces Broader Class Switching but Fails to Increase the Functional Avidity of the Antibody Response

To assess the functionality of Env-specific antibodies elicited by the various immunogens, the Env-specific avidity and antibody-class switching was assessed using pooled sera from animals in each group that showed above-threshold titers to AD8 SOSIP at week 16. Avidity ELISAs showed that the groups that were vaccinated with iVLP SOSIP, mVLP SOSIP, ΔVLP SOSIPHA, or mVLP SOSIPHA all displayed similar avidity for the SOSIP gp140 antigen, as demonstrated by the similar concentrations (1–1.5 M) of sodium thiocyanate (NaSCN) needed to reduce antibody binding by half (Figure 6D). In contrast, the SOSIP gp140-vaccinated group showed greater avidity for the antigen.

The degree of antibody-isotype switching was measured as a proportion of the total antibody response. The iVLP SOSIP- and ΔVLP SOSIPHA-vaccinated groups exhibited Env-specific antibody-isotype distributions that were distinct from each other and narrower than other groups: iVLP SOSIP-vaccination gave rise to mostly IgG2a responses and slightly less IgG2b responses, while vaccination with ΔVLP SOSIPHA elicited an even proportion of IgG1 and IgA anti-Env antibody titers (Figure 6E). Animals vaccinated with SOSIP gp140 or mVLP SOSIP demonstrated a broader distribution of Env-specific antibody-isotype responses, as shown by a roughly equal contribution from IgG1, IgG2a, IgG2b, and IgA isotypes. Animals that received mVLP SOSIPHA showed a similarly broad antibody-isotype response with additional Env-specific IgG3 responses that were not observed in any other vaccination group. IgM isotype responses were also assayed, but no response above the threshold was detected for any vaccination group.

### 3.9. VLP Vaccinations Elicit Anti-Gag Antibody Responses That Do Not Negatively Impact Env-Specific Responses

**Figure 7:**
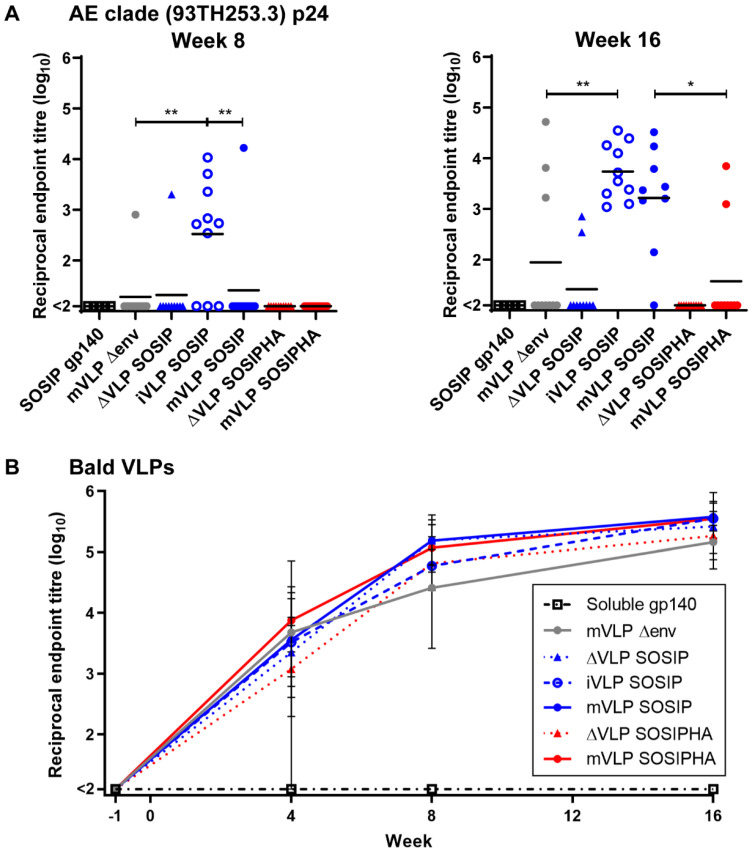
Mouse serum Gag-specific and Bald VLP-specific antibody responses. (**A**) Reciprocal endpoint serum antibody titers (log_10_-transformed) at week 8 and 16 against AE clade (93TH253.3 strain) p24 as determined by p24-capture ELISA. Each point represents an individual animal. The black bar represents the mean titer. The endpoint dilution titer cut-off was defined as 3 times the average OD measured for the same serum dilution of adjuvant-only-vaccination group mice sera sampled at the same time point. Significance was determined by a Kruskal–Wallis test followed by Dunn’s multiple comparison test. *p*-values are indicated as *p* < 0.05 (*), < 0.01 (**). Multiple comparisons were performed as follows: mVLP Δenv vs. iVLP SOSIP, mVLP Δenv vs. mVLP SOSIP, mVLP Δenv vs. mVLP SOSIPHA, iVLP SOSIP vs. mVLP SOSIP, and mVLP SOSIP vs. mVLP SOSIPHA. (**B**) Reciprocal endpoint serum antibody tires (log_10_-transformed) at weeks –1, 4, 8, and 16 against Bald VLPs (mVLP Δenv) as determined by VLP ELISA. Each point represents the mean, and the error bars show the range of titers for all animals within the vaccination group. The endpoint dilution titer cut-off was defined as 3 times the average OD measured for the same serum dilution of adjuvant-only-vaccination group mice sera sampled at the same time point. For both (**A**,**B**), the lowest reciprocal dilution assayed was 100, and titers less than 100 were recorded as “<2”. Data are representative of 2 independent experiments.

The humoral responses to internal VLP proteins were investigated by assessing the reactivity of individual animal sera against AE clade p24 (93TH253.3 strain, used to generate the iVLPs and mVLPs), which was captured by a p24-specific mAb. At week 8, most animals in the iVLP SOSIP group showed modest p24-specific titers ranging between 1:345 and 1:10,666, and a single animal in each of the mVLP Δenv and mVLP SOSIP groups showed above-threshold titers within the same range (Figure 7A). The iVLP SOSIP-vaccination group titers were significantly higher than either the mVLP Δenv- or mVLP SOSIP-group titers, due to most animals in the latter two groups not having above-threshold titers. A single animal from the ΔVLP SOSIP-vaccinated group also exhibited an apparent above-threshold anti-p24 titer, which was surprising given the ΔVLP expression plasmids do not encode p24. As the p24 antigen used was derived from solubilized mVLP Δenv particles, this suggested there may have been other cellular proteins associated with the captured p24 that were recognized by the serum antibodies of this animal. The antibody responses at week 16 were increased for iVLP SOSIP-vaccinated animals, and all animals within this group showed above threshold titers. The responses were significantly higher than those seen for mVLP Δenv-vaccinated animals, with only three animals in this group displaying any anti-p24 titers above background. There was an increased number of animals vaccinated with mVLP SOSIP that exhibited p24-specific titers above threshold and these were significantly higher than titers seen for mVLP SOSIPHA-vaccinated animals, with two animals in this group having titers above background. Two animals from the ΔVLP SOSIP group appeared to have detectable anti-p24 titers at week 16, albeit ~10-fold lower than the average titers observed in iVLP or mVLP groups, but this was likely due to cellular contaminants, as described previously. As expected, no responses were seen from any mice in the SOSIP gp140 or ΔVLP SOSIPHA groups. Finally, to assess whether p24 antibody responses may have enhanced or rendered Env responses subdominant, Spearman’s rank-order correlation testing was conducted for iVLP SOSIP-, mVLP SOSIP-, and mVLP SOSIPHA-vaccinated animals. The differences in p24-specific antibody responses between these groups did not appear to have any influence on the generation of SOSIP gp140-specific antibody responses (Figure S7A). Similarly, no significant correlation was observed between anti-p24 antibody responses and UNC gp140-specific antibody titers for animals vaccinated with iVLP SOSIP or mVLP SOSIP (Figure S7B). The mVLP-SOSIP-vaccinated group showed a significant positive correlation between p24- and UNC-gp140-specific antibody responses; however, this result may not be biologically relevant, given only two out of 10 animals had above-threshold anti-p24 antibody titers.

### 3.10. Humoral Responses in Mice Following VLP Vaccination Are Largely Directed against Human Antigens Presented on the VLP Surface

The use of human-cell-produced VLPs in nonhuman animal-vaccination studies had previously been reported to induce antibodies targeting non-HIV-1 proteins [101,102], and this was expected to occur with the VLPs and mouse model used here. To measure the level of anti-human-lipid and -protein humoral responses, individual animal sera from all bleed collection time points were assayed for reactivity against mVLP Δenv (Bald VLPs) by VLP ELISA. The magnitude of antibody binding for all animals was close to the assay background prior to vaccinations (week −1), which suggested there were no inherent anti-human-antibody responses in the animal sera. As expected, animals from the AD8 gp140-vaccinated group did not develop any antihuman titers over the course of the vaccination regime (Figure 7B). In contrast, almost all animals that received Env-bearing extracellular vesicles, iVLPs, or mVLPs (with the exception of one animal in the ΔVLP SOSIPHA-vaccination group) showed above-threshold Bald-VLP-specific antibody titers at week 4 that ranged between 1:198 and 1:71,614. These titers were higher at week 8, with all animals from these groups showing above threshold titers. At week 16 the anti-Bald-VLP titers showed a further slight increase and were relatively similar amongst all animals in these groups, ranging between 1:53,088 and 1:394,457. To determine whether the presence of Bald-VLP-specific humoral responses had any impact on the Env-specific antibody titers, Spearman’s rank-order correlation testing was used for Bald-VLP titers versus SOSIP gp140 or UNC gp140 titers as measured at week 16 (Figure S7C, closed and open circles, respectively). Analysis with animals from the ΔVLP SOSIP, iVLP SOSIP, mVLP SOSIP, ΔVLP SOSIPHA, and mVLP SOSIPHA groups showed no significant correlation for responses to either Env antigen. The correlation between antibody responses to Gag (p24) and Bald VLPs for animals within the mVLP Δenv, iVLP SOSIP, mVLP SOSIP, and mVLP SOSIPHA groups at the same week also did not demonstrate significant correlation (Figure S7D).

### 3.11. Limited Neutralizing Antibody Responses Are Detected after mVLP SOSIPHA Vaccination of Mice

In order to determine whether antibodies capable of neutralizing HIV-1 virions were elicited, sera from animals at week 16 that displayed above-threshold Env-specific titers (to either SOSIP gp140 or UNC gp140) were pooled according to the vaccination groups for use in neutralization assays. Additionally, sera from all animals in the adjuvant-only and mVLP Δenv groups were pooled for use in these assays to measure nonspecific neutralization. Despite the presence of antibodies in VLP-vaccinated animal sera that targeted human proteins and lipids presented on the VLP surface, the human-origin TZM-bl cell line was used for neutralization assays, since it had been previously shown that antihuman responses did not result in significant nonspecific neutralization with this cell line [102]. Neutralization was measured against pseudoviruses expressing the AD8 vaccination strain (a difficult-to-neutralize, tier 3 isolate [103]) and two tier 1 viruses, MN and SF162. Neutralization against control virus pseudotyped with the amphotropic MuLV Env was also assessed for all groups to confirm there was no nonspecific neutralization of pseudovirus. As expected, the adjuvant-only and mVLP Δenv groups did not reach neutralization ID50 at the lowest dilution tested, 1:25 (Table 4). The SOSIP gp140-vaccinated animals showed a strong neutralization ID50 titer of 1:8625 against MN and a lower titer against SF162 (1:59). The mVLP-SOSIPHA-vaccinated animals demonstrated an approximately 100-fold lower neutralization ID50 titer against MN virus (1:82), but the neutralization ID50 against SF162 was less than 1:25. No other vaccination groups achieved neutralization ID50 at the lowest dilution tested.

## 4. Discussion

In this study, mutations and substitutions within the HIV-1 Env TM and CT domains that were made with the aim of increasing Env trimer incorporation into previously designed mature-form HIV-1 VLPs [57] were investigated. The amount of gp41 incorporated into mVLPs was enhanced when the TM and CT domains of cleavage-competent Env (with or without an SOS modification) were substituted with the analogous domains from the MMTV *env* gene or IFA HA (MMTV-TMCT and HA-TMCT, respectively). These substitutions more efficiently enhanced gp41 incorporation than removal of N- and C-terminal endocytosis motifs within the gp41 CT (Δendo) or deletion of the entire CT domain (ΔCT), which were based on previously employed strategies to increase Env surface expression on transfected cells [86,87,91] or enhance the incorporation of Env into viral particles [92]. Most studies of Δendo and ΔCT modifications have utilized viral particle expression vectors derived from B clade viral isolates, whereas the *gag* and *pol* sequences of the mVLP expression vector used here are AE-clade-derived, thus for the Δendo Env, the minimal incorporation enhancement may be due to differences in the interaction between the B-clade CT and AE-clade Gag polyprotein. For the ΔCT, where interactions between gp41 and Gag are not expected, the reasons for poor Env incorporation are unclear. Despite TM and CT substitutions yielding enhanced gp41 incorporation, increases in gp120 loading onto mVLPs were not concomitantly observed relative to the wild-type Env controls. While this phenomenon was not investigated in detail here, the tendency for gp120 to dissociate from the gp41 domain (gp120 shedding) [26,27] was likely occurring from Env expressed on the surface of VLP-transfected cells or from Env on the surface of particles following membrane budding and scission regardless of the presence of the SOS modification. This was somewhat counterintuitive, given the SOS mutation can create a covalent bond between gp120 and gp41 domains that should be resistant to shedding, but less surprising since the disulfide bond does not necessarily form with high efficiency [80], and the efficiency would be expected to be strain-dependent. It was also interesting that the two TM/CT substitutions influenced gp120 incorporation differently: the MMTV-TMCT-modified cleavage-competent Env showed reduced gp120 loading when compared to HA-TMCT-modified sequences, even though the gp41 incorporation was similar. Given the HA-TMCT substitution was shown to affect gp41 conformation by its ablation of the mAb Z13e1 binding site within the MPER, these observations suggest the alternative TM/CT domains imposed differing conformational changes to the gp41 domain that may reduce the formation and/or stability of the noncovalent interactions between gp41 and gp120. Furthermore, these differences in gp120 shedding are consistent with previously published findings that mutations within gp41 affecting its conformation can negatively impact the ability of gp120 to be retained on the virus surface [104].

To prevent gp120 shedding, the removal of the gp120-gp41 cleavage site in combination with the TM and CT modifications was utilized, and this facilitated increases in Env incorporation of between 10- and 30-fold relative to uncleaved Env without TM or CT modifications. The enhanced incorporation of MMTV-TMCT- and HA-TMCT-modified Env were broadly consistent with the improved incorporation reported when the same substitutions were used with insect-cell-produced HIV-1 VLPs [33], although the HA-TMCT enhanced incorporation more efficiently in this study. These results also contrasted with a study incorporating similar Env/HA-TMCT constructs into Gag-only VLPs that showed no improvement over wild-type Env [105]. These differences are probably reflective of the different cell lines and VLP expression vectors used. Given the superior incorporation of uncleaved Env modified with the HA-TMCT, inserting a flexible amino-acid linker sequence between the uncleaved gp120 and gp41 domains was investigated as a method to mimic the antigenicity of a cleaved trimeric Env, as had been demonstrated previously for soluble Env proteins [94]. None of the linker sequences, either with or without an SOS-only mutation, improved Env antigenicity, but the introduction of 10 or 15 amino-acid linkers to an SOSIP-stabilized membrane-bound Env was effective at enhancing the proportion of compact, trimeric (native-like) Env on the surface of mVLPs, as demonstrated by improved binding to VRC26.06 relative to wild-type Env. These observations indicated the I559P substitution was critical for the enhanced trimer presentation. The 10-amino-acid linker (L10) was more effective at enhancing VRC26.06 binding than a 15-amino-acid length sequence (L15); however, much greater binding was seen with no flexible linker sequence between the uncleaved domains (L0), which was an almost inverse result to that reported for soluble, single-chain Env proteins [94]. This suggested the conformation of flexible linker-stabilized, membrane-anchored Env with the I559P mutation was significantly different to soluble forms of similarly mutated Env, and this was supported by assessment of the antigenicity of soluble gp140 equivalents of the linker-stabilized Envs that showed L0 Envs were unable to bind VRC26.06, while small increases in binding were measured for L15 and L10 within SOSIP gp140. The unexpected results for membrane-bound Env suggest further study into whether alternative linker lengths or stabilizing mutations can be used with flexible amino-acid linkers to maintain a compact trimeric Env structure, including the use of both longer and shorter linkers, is warranted. Additionally, the need to test the I559P mutation in isolation, given it has been previously implicated in altering the conformation and epitope presentation of membrane-bound Env [99], is indicated.

The improved presentation of native-like trimers on mVLPs using the L0 SOSIP Env was superseded by the cleavage-competent, SOSIP-stabilized Env (WT SOSIP), which was initially included as a control for assessing the flexible linker-stabilized Envs. The WT SOSIP demonstrated slightly greater presentation of native-like trimers, as demonstrated by VRC26.06 binding, and bound many of the bNAbs assessed with the highest or generally good affinity. In particular, it bound PGT151 more strongly than wild-type Env and with far greater efficiency than L0 SOSIP, which was expected given the lack of a functional cleavage site in the latter construct [68]. Because of the broadly enhanced bNAb binding and reduced non-neutralizing antibody binding, the WT SOSIP Env was utilized for further characterization in combination with the HA-TMCT substitution that had facilitated increased incorporation of Env into the mVLPs. The HA-TMCT sequence did not have a major impact on the conformation of bNAb epitopes, as demonstrated by no loss of binding to the panel of bNAbs assessed, including the MPER bNAb 10E8, but with the exception of Z13e1 as mentioned earlier. In addition to partly altering the conformation of the MPER, the HA-TMCT substitution also ablated the fusion activity of HIV-1 Env, as assessed by fluorescent fusion assays. The SOSIP mutations used in the VLP immunogens inhibit the fusion activity of Env [97,98,99], and the HA-TMCT provides an additional fusion inhibition mutation that contributes towards the safety profile of VLPs bearing this Env, as well as enhancing Env incorporation. Given the HA-TMCT mutation to improve Env incorporation was screened in the context of a wild-type, SOS, and uncleaved Env ectodomain, further screening could reveal TM/CT modifications that specifically enhance incorporation of the SOSIP Env. Future work could reassess the other TM/CT modifications tested here or alternative modifications such as TM/CT sequences derived from different isolates of IFA HA or a wider range of viral glycoproteins.

Expression of VLPs from a single plasmid vector was found to result in an increased amount of Env-containing microvesicles and/or exosomes when compared to VLPs expressed from separate particle- and Env-expressing vectors. This was observed for both Env with wild-type TM/CT sequences and heterologous sequences from IFA HA, and in both adherent 293T and suspension Expi293F expression systems. A likely source of these contaminants are exosomes, given their similar buoyant density to HIV-1 viral particles [106] and previous observation to not be separated from virions via sucrose gradients or cushions [107]. Alternatively, these extracellular vesicles could be composed of microvesicles, which are typically larger (>200 nm) than HIV-1 virions [108], or the generally smaller nanovesicles that are likely derived from the plasma membrane [109]. Indeed, the expression of contaminants is likely related to Env expression levels, as suggested by the low proportion of non-VLP particles seen in VLP cotransfection where Env expression is reduced, and the increased contamination when VLPs expressed more Env due to the HA-TMCT modification. Further experiments would be needed to determine whether the effect is strictly due to Env expression or associated with the strain of Env (and Gag or Pol) used in these studies. Characterization of extracellular vesicles coexpressed in HEK cells once transfected with HIV-1 VLP expression vectors has shown the act of transfection is sufficient to induce morphological changes in these particles [110]. Nonetheless, these observations highlight the need for more efficient and scalable methods for the removal of contaminating particles from HIV-1 VLP immunogens in the production of potential VLP vaccines. An already-established method for removal of contaminating particles from virus preparations is by CD45 immunodepletion [111,112], which is possible given cellular CD45 is excluded from HIV-1 particles [113]. However, since CD45 is not expressed by the nonhematopoietic (i.e., 293T and Expi293F) cell lines typically used for large-scale expression [113,114,115], this strategy would require the generation of novel cell lines bearing CD45 on their surface. A more recent advance in VLP purification techniques is the use of AEX or hydroxyl-functionalized polymethacrylate monoliths that separate VLPs from contaminants (and even dsDNA) based on particle charge and hydrophobicity [116,117]. Heparin-affinity chromatography has also been shown to facilitate high-resolution separation of VLPs and cellular particles along with host-cell chromatin [118]. These kinds of technology are likely to facilitate the preparation of high-purity mammalian cell-derived VLPs that may be immunogenically superior to those resulting from existing techniques.

The VLP immunogens were less efficient at eliciting Env-specific antibodies when compared to mice vaccinated with the soluble Env protein (SOSIP gp140). This was demonstrated by the generally sporadic induction of humoral responses and the generally lower antibody titers resulting from VLP vaccination. Furthermore, the difference in the quality of the response to VLP and soluble immunogens was highlighted by the enhanced Env-specific antibody avidity exhibited by SOSIP gp140-vaccinated mice as compared to the VLP-vaccinated mice. The antibody responses to VLP-associated Env were possibly limited by the substantial humoral response targeting human proteins and lipids (as illustrated by consistently robust anti-Bald-VLP titers for all VLP groups) that arose following the first vaccine dose. However, no negative correlation between Bald-VLP- and Env-specific antibody titers was found, suggesting anti-Env responses were not limited stochastically. Alternatively, these early anti-human-antibody responses may have facilitated enhanced antibody-mediated clearance of subsequent VLP doses, thereby reducing the amount of VLP-associated Env available to stimulate B cells. Responses to the internal VLP proteins as measured by anti-p24 endpoint titers were similarly not negatively associated with higher anti-Bald-VLP titers. Nonetheless, such cross-species responses are common when vaccinating nonhuman species with VLPs produced in human cell lines [101,102,119,120], and this effect is compounded by the limited immunogenicity of Env. This is especially true given the vast array of human cellular proteins populating the surface of HIV-1 viral particles [111,113,121]. It should be noted that when VLPs are produced in human cells, a human vaccine recipient is not expected to elicit an immune response against these non-HIV-1 antigens, potentially facilitating a stronger anti-HIV-1 response. Further experiments to assess the immunogenicity of VLPs in nonhuman animals would benefit from using VLPs generated in cell lines that match the species of the animals being used for vaccination, for example by using nonhuman primate cell line produced VLPs to immunize nonhuman primate animals. However, beyond primates, this would be difficult with the VLP expression cassettes used here, given the expression of HIV-1 structural proteins (*gag*, *pol*, and *env*) is regulated by Rev, which is generally unable to efficiently coordinate mRNA export in rodent cells [122]. The alternative approach of expressing the VLPs in mice or rats in vivo via a DNA vaccine vector would also be prone the same the Rev restriction. Studies using in vivo- or in vitro-produced VLPs using rabbits or rabbit cell lines, respectively, may be effective given rabbit cells do permit some HIV-1 replication and, in particular, do not limit Rev function [123]. Additionally, rabbits are more capable than rodents at eliciting neutralizing antibody responses against Env [51,102,124] due to their antibody repertoire having longer CDRH3 sequences, which are often found in bNAbs [125]. The cost and availability of rabbits for sufficiently powered vaccination studies is prohibitive when compared to mice or rats.

For most of the VLP-vaccination groups, the small number of animals that generated Env-specific titers above the assay threshold limited the meaningful statistical analysis that could be conducted; the majority of significant differences observed were predominantly comparisons with groups within which the majority of animals displayed titers <1:100. Env-specific titers were determined by assessing antibody binding to a soluble SOSIP gp140, given the same modifications were used within the VLP-anchored Env, and to an uncleaved gp140 since a proportion of the Env incorporated into the VLPs was uncleaved, which is typical for HIV-1 viral particles [9]. Of the animals that mounted anti-Env humoral responses to both antigens, there were similarly positive correlations of endpoint titers regardless of the immunogen used. One point of interest was that mVLP-SOSIPHA-vaccinated mice demonstrated responses that were significantly higher than mVLP-SOSIP-vaccinated mice against uncleaved Env, but not against SOSIP gp140. This suggested the mVLP SOSIP particles may have more efficiently elicited antibody responses to nativelike Env epitopes than to epitopes exclusive to uncleaved Env. This was surprising, since the mVLP SOSIP and mVLP SOSIPHA particles demonstrated similar binding profiles for quaternary Env epitope-directed mAbs, such as PGT145 and PG9, which are better presented by native-like trimers [126,127]. Furthermore, the mVLP SOSIPHA appeared to have higher Env cleavage as shown by a small increase (~3-fold) in binding of the cleavage-dependent PGT151 [68]. The specificity of the mVLP SOSIP and mVLP SOSIPHA vaccinal sera could be further investigated using a panel of Envs with alanine point mutations as described previously [51]. However, the volume of sera required for such an analysis would necessitate an immunization study in larger animals such as rabbits or macaques. Additionally, polyclonal sera may be too broad in the exact epitopes targeted for a given conserved Env site for point mutations to reveal a significant change in binding.

When assessing the anti-Env antibody isotype responses following vaccination with mVLPs, there were noticeable differences relative to the responses generated by either iVLPs or ΔVLPs. The Env-specific antibody-class switching resulting from mVLP vaccination was broad, with IgG1, IgG2a, IgG2b, and IgA responses detected. This was in stark contrast to mice receiving either iVLPs or ΔVLPs, where the class switching was limited to IgG2a/IgG2b or IgG1/IgA responses, respectively, that would restrict the potential Env-specific antibody effector functions [128]. This change is likely due to differences in Env presentation on the surface of mVLPs whereby mature HIV-1 virions allow Env to laterally translocate and cluster on the particle surface [58]. Indeed, clustering of Env on mVLPs would be expected to better stimulate B cells via multivalent presentation of Env epitopes to facilitate enhanced antibody receptor cross-linking [129,130,131]. It is important to note, however, that SOSIP gp140, being a soluble protein immunogen, was not expected to efficiently present multivalent epitopes, but nevertheless showed similarly broad antibody-class switching. It was also interesting that the mVLP-SOSIPHA-vaccinated mice showed Env-specific IgG3 antibody responses in addition to the broad range of isotypes already mentioned. The high density of Env resulting from HA-TMCT modification may have better induced Th1 cell responses and subsequent interferon gamma expression [132,133]. This could be determined in further vaccination studies by analyzing the T helper cell phenotype and cytokine profile of Env-reactive CD4+ T cells following immunization with high- and low-density Env-bearing VLPs.

In addition to the differences between mVLP- and iVLP-vaccinated mice in relation to Env-specific antibody-class switching, the humoral response to internal VLP proteins were significantly different. Most of the animals vaccinated with iVLPs demonstrated above-threshold anti-p24 titers at week 8, whereas most mVLP-vaccinated animals had undetectable responses. This is consistent with the enhanced Gag-specific cytotoxic T-cell responses reported for mice vaccinated with immature Gag-only particles versus those receiving particles composed of Gag and Pol resembling mature virions [134]. At the later time point of week 16, there was no significant difference between iVLP SOSIP and mVLP SOSIP anti-p24 titers, but the mVLP SOSIP titers were significantly higher than those seen in mVLP-SOSIPHA-vaccinated mice. However, this is most likely a reflection of the different dose of mVLPs used for these two groups: the mVLP SOSIPHA vaccine doses contained fewer particles and therefore less p24 antigen, since the incorporation of Env was higher in the presence of the HA-TMCT modification. It was also interesting to note only 3 of the 10 mice in the control group that received the Env-negative mVLP (mVLP Δenv) demonstrated p24-specific antibody titers above the threshold, despite being vaccinated with the same quantity of p24 as used for mVLP SOSIP-vaccinated mice where 9 of the 10 mice responded. This implied the membrane-bound Env played a role in facilitating humoral responses to the normally membrane-occluded Gag protein. However, this is unlikely given the lack of meaningful correlations between p24 antibody titers and antibody responses against either SOSIP or uncleaved gp140. Additionally, 1 animal at week 8 and 2 animals at week 16 vaccinated with ΔVLP SOSIP, which did not contain p24 antigen, displayed measurable titers. This was likely caused by reactivity to cellular proteins associated with the captured p24 in ELISAs. Therefore, it is possible that some of the other measured p24 titers may in fact be due to reactivity with these cellular proteins. To resolve this issue, future experiments could employ instead a purified recombinant AE-clade p24 protein, which was not available for the experiments conducted here.

VLP-vaccinated mouse sera generally showed no neutralizing activity, except for some modest neutralization of the tier 1A MN strain virus by mVLP-SOSIPHA-vaccinated mice. Given mice that were vaccinated with SOSIP gp140 generated higher titers of antibodies that were able to neutralize both MN and the tier 1B SF162 virus, this suggests the generally lower Env-specific antibody titers induced by VLP vaccination were not high enough for neutralization to be detected with the assay protocol used. Nevertheless, it is important to note the mVLP-SOSIP-vaccinated animals that elicited Env-specific antibodies did not show any neutralizing activity at the lowest dilution tested despite having similar endpoint titers to SOSIP gp140 or mVLP-SOSIPHA-vaccinated mice. This suggests the higher Env density on HA-TMCT-modified mVLPs was able to induce better neutralizing antibody activity. The increased surface Env density was approximately 2.5-fold for mVLP SOSIPHA relative to mVLP SOSIP as based on the average ratio of Gag (p24) and Env (gp120) concentrations for these VLPs (18.8 versus 7.42 trimers per VLP, using the assumption that 2000 Gag molecules form a particle [135] and all Env are trimeric). While these estimates are in the range of estimations of Env per virion reported for certain HIV-1 isolates [28,29,30,31,58], the quantitation of Gag (and therefore particle number) is not absolute, given measurement was performed using standards and reagents made using B-clade protein, whereas the VLP Gag is derived from an AE-clade sequence. However, the difference between SOSIP and SOSIPHA mVLPs should hold true, given the Gag was identical between these samples.

## 5. Conclusions

Chimeric TM/CT domains based on IFA HA were found to effectively improve Env incorporation into HIV-1 mVLPs without significant impairment of bNAb epitope presentation. The introduction of SOSIP mutations stabilized the incorporated Env and enhanced the display of quaternary bNAb epitopes. The presentation of membrane-bound Env resembling functional viral Env on HIV-1 VLPs is a useful approach for the induction of HIV-1-specific antibody responses. Unfortunately, the generation of antibodies targeting the human proteins and lipids that were abundant on the VLPs may have dampened the immune response against HIV-1 antigens, limiting the immunogenic inferences that could be made between different particle morphologies and incorporated Env densities. The mature VLPs did show broader Env-specific antibody responses, which is a desirable outcome given polyfunctional anti-Env antibody responses have been implicated in the protective benefit afforded to vaccine recipients in the RV144 trial [136]. Therefore, VLPs resembling mature HIV-1 virions are promising immunogens for HIV-1 Env and warrant further investigation, especially when expressed in vivo from nucleic acid vaccine vectors.

## Figures and Tables

**Table 1:** Fold-change in mAb binding to mVLP-associated linker-stabilized Env.

mAb	PBS Only	Env Only	Bald	WT	WT SOS	WT SOSIP	L0	L0 SOS	L0 SOSIP	L10	L10 SOS	L10 SOSIP	L15	L15 SOS	L15 SOSIP
2G12	0.03	0.31	0.04	1	1.00	1.12	0.90	1.10	1.11	0.92	1.04	1.12	0.86	1.07	1.13
2F5	0.06	0.24	0.06	1	1.96	1.88	1.02	2.12	2.13	0.90	1.87	2.46	0.97	2.00	2.42
VRC01	0.02	0.33	0.02	1	1.09	1.21	0.97	1.05	1.23	0.73	0.96	1.20	0.72	0.96	1.15
VRC26.06	0.20	0.41	0.22	1	1.16	3.62	0.64	0.71	2.96	0.66	0.72	1.74	0.69	0.64	1.47
PGT121	0.02	0.30	0.02	1	1.11	1.48	0.91	0.97	1.47	0.63	0.80	1.33	0.55	0.78	1.21
35O22	0.11	0.20	0.12	1	1.87	4.84	0.57	1.78	4.38	0.59	1.66	4.42	0.58	1.61	3.78
PGT151	0.11	0.21	0.11	1	1.79	1.33	0.24	0.26	0.40	0.25	0.24	0.67	0.24	0.22	0.67
10E8	0.02	0.05	0.02	1	0.86	0.84	0.73	0.99	0.89	0.62	0.86	1.12	0.75	0.93	1.09
F105	0.02	0.21	0.02	1	1.11	0.86	1.12	1.26	0.93	0.91	1.11	1.09	0.80	1.17	1.15
17b	0.04	0.26	0.04	1	2.28	2.13	1.23	1.00	2.27	0.92	0.91	3.35	0.84	0.88	3.46
447-52D	0.01	0.41	0.01	1	1.01	0.91	0.96	0.98	0.93	0.74	0.85	0.88	0.66	0.82	0.88
F240	0.01	0.29	0.01	1	0.16	0.04	0.81	0.20	0.04	0.65	0.16	0.04	0.68	0.15	0.03

**Table 2:** Fold-reduction in bNAb binding for ΔVLPs expressing SOSIP or SOSIPHA Env.

bNAb	ΔVLP SOSIP vs. mVLP SOSIP	ΔVLP SOSIPHA vs. mVLP SOSIP
VRC01	2.0	1.5
PG9	3.7	2.2
PGT145	3.3	1.7
2G12	1.9	1.5
PGT121	3.5	1.7
35O22	5.4	1.7
PGT151	14.6	2.8
10E8	3.4	1.5

Area under the curve (AUC) was calculated from mean ELISA binding curves shown in Figure 5. Fold-reduction was calculated by dividing the AUC for the ΔVLP by the appropriate mVLP sample.

**Table 3:** Mouse vaccination dose normalization.

Immunogen	Normalization
Adjuvant only	N/A
SOSIP gp140	Env
mVLP Δenv	Gag normalized to mVLP SOSIP
ΔVLP SOSIP	Volume of iVLP/mVLP SOSIP (largest volume)
iVLP SOSIP	Env
mVLP SOSIP	Env
ΔVLP SOSIPHA	Volume of mVLP SOSIPHA
mVLP SOSIPHA	Env

**Table 4:** Reciprocal ID50 serum neutralization titers.

	Reciprocal ID50 Serum Titers ^a^		
Immunogen	MN	SF162	AD8
Adjuvant only	<25	<25	<25
SOSIP gp140	8625	59.2	<25
mVLP Δenv	<25	<25	<25
ΔVLP SOSIP	<25	<25	<25
iVLP SOSIP	<25	<25	<25
mVLP SOSIP	<25	<25	<25
ΔVLP SOSIPHA	<25	<25	<25
mVLP SOSIPHA	81.7	<25	<25

^a^ Defined as the average pooled sera dilution that inhibited 50% of viral infectivity in TZM-bl cells (*n* = 2). Uncolored values display no measured neutralization (<25), yellow values show moderate neutralizing activity (25–100), and red values display high neutralizing activity (>1000).

## Data Availability

No new data were created or analyzed in this study. Data sharing is not applicable to this article.

## Supplementary Materials

The following are available online at https://www.mdpi.com/2076-393X/9/3/239/s1, Figure S1: Genetic organization of linker-stabilized and cleavage-competent Env sequences and quaternary Env epitope presentation by soluble linker-stabilized gp140 proteins, Figure S2: Genetic organization of ΔVLP vector and level of Env presented on mVLPs and ΔVLPs, Figure S3: 293T and Expi293F per-particle Env antigenicity, and comparison of 293T- versus Expi293F-produced particle-associated Env antigenicity, Figure S4: Assessing decreased transfection plasmid copy number for reduction of microvesicle- and exosome-associated Env from VLP preparations, Figure S5: Correlation of UNC and SOSIP Env responses, Figure S6: Correlation of Env, p24, and Bald VLP mouse serum antibody responses, Figure S7: Uncropped images of Western blots from Figure 1B,C, Figure S8: Uncropped images of Western blots from Figure 2B,C.
