# Supplementary material for: Immunogenicity of HIV-1-Based Virus-Like Particles with Increased Incorporation and Stability of Membrane-Bound Env
Source: Vaccines (Basel). 2021 Mar 10;9(3):239. doi: 10.3390/vaccines9030239 (PMC8002006; doi:10.3390/vaccines9030239)
Supplement: Supplementary file 1 [file vaccines-09-00239-s001.pdf]

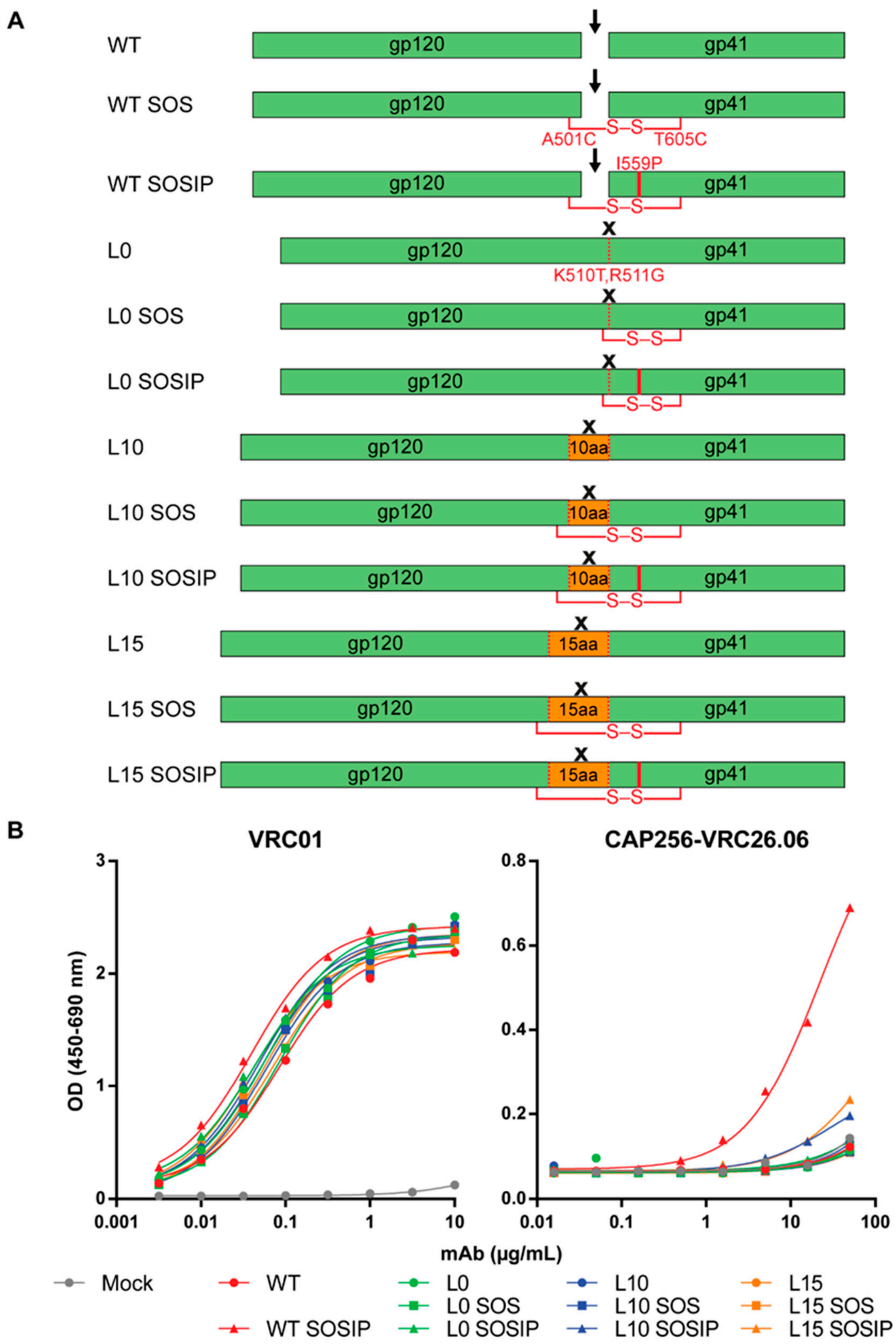

Figure S1. Genetic organization of linker-stabilized and cleavage-competent Env sequences and quaternary Env epitope presentation by soluble linker-stabilized gp140 proteins. (**A**) Schematic representation of uncleaved AD8 gp160 constructs with zero (UNC), 10 (L10) or 15 (L15) serine-glycine amino acid linker sequences inserted between the gp120 and gp41 domains. Also shown are gp160 sequences with a functional gp120-gp41 cleavage site (WT). Where indicated, linker-stabilized and cleavage-competent gp160 sequences also contained a SOS modification in isolation (SOS), or in combination with an I559P amino acid substitution within gp41 (SOSIP). (**B**) Representative ELISA binding curves (from two independent experiment repeats) of VRC01 and CAP256-VRC26.06 to soluble Env captured from cell culture supernatant via a D7324 antibody epitope tag following transfection with linker-stabilized and cleavage-competent gp140 expression vectors containing SOS or SOSIP modifications. Supernatant from cells transfected with an empty vector was used as the “Mock”. The volume of supernatant loaded in each well was equal for each construct.

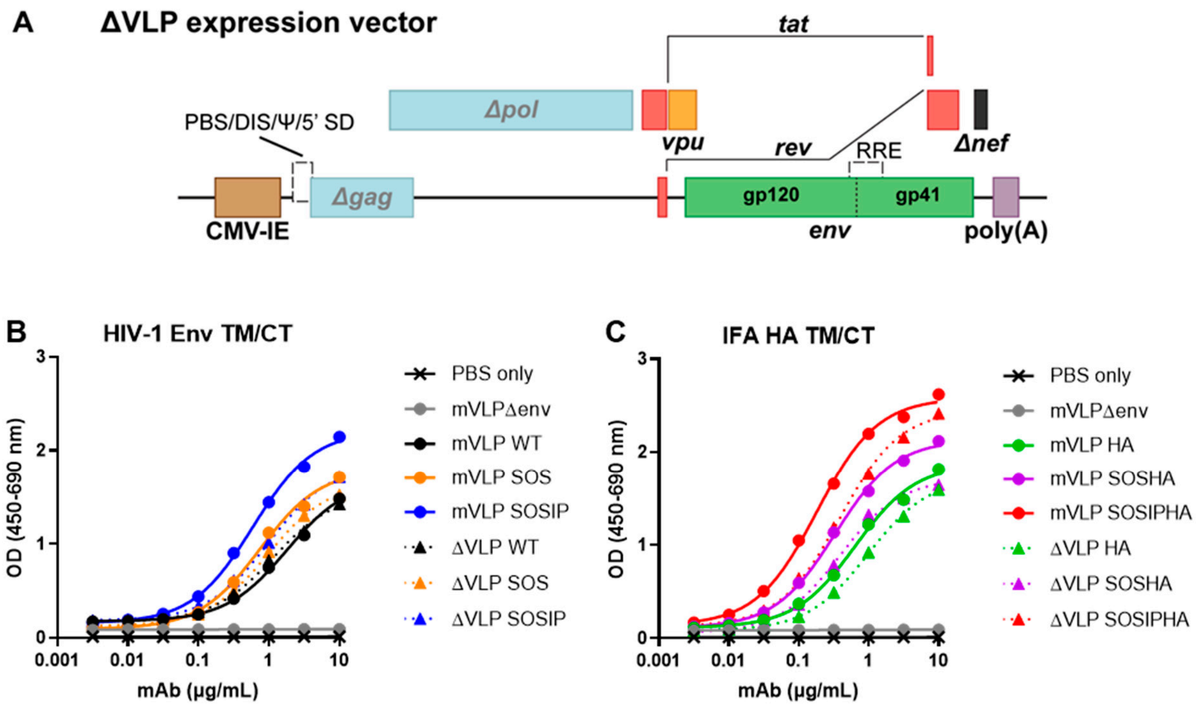

Figure S2. Genetic organization of ΔVLP vector and level of Env presented on mVLPs and ΔVLPs. (**A**) Schematic representation of pCMV-AEBΔ17MΔ24 (ΔVLP) expression plasmid. The gag gene is defective due to removal of potential start codons within the p17 domain reading frame, deletion of the middle half of the p24 domain, and introduction of a premature stop codon within Gag (M142*), ablating pol gene expression. CMV-IE: human cytomegalovirus immediate-early promoter, PBS: primer binding site, DIS: dimerization initiation sequence, Ψ: packaging signal, 5′ SD: 5′ splice donor site, RRE: Rev-responsive element. (B) Env with an HIV-1 Env TM/CT or (**C**) Env with an IFA HA TM/CT were assessed by VLP ELISAs performed using an equal number of particles/well as determined by p24 ELISA for mVLP samples. Env modifications are equivalent to those described in Figure 3A. ΔVLP samples were volume equalized to the mVLP sample encoding the same Env modification. Representative ELISA data shown from two independent experiments.

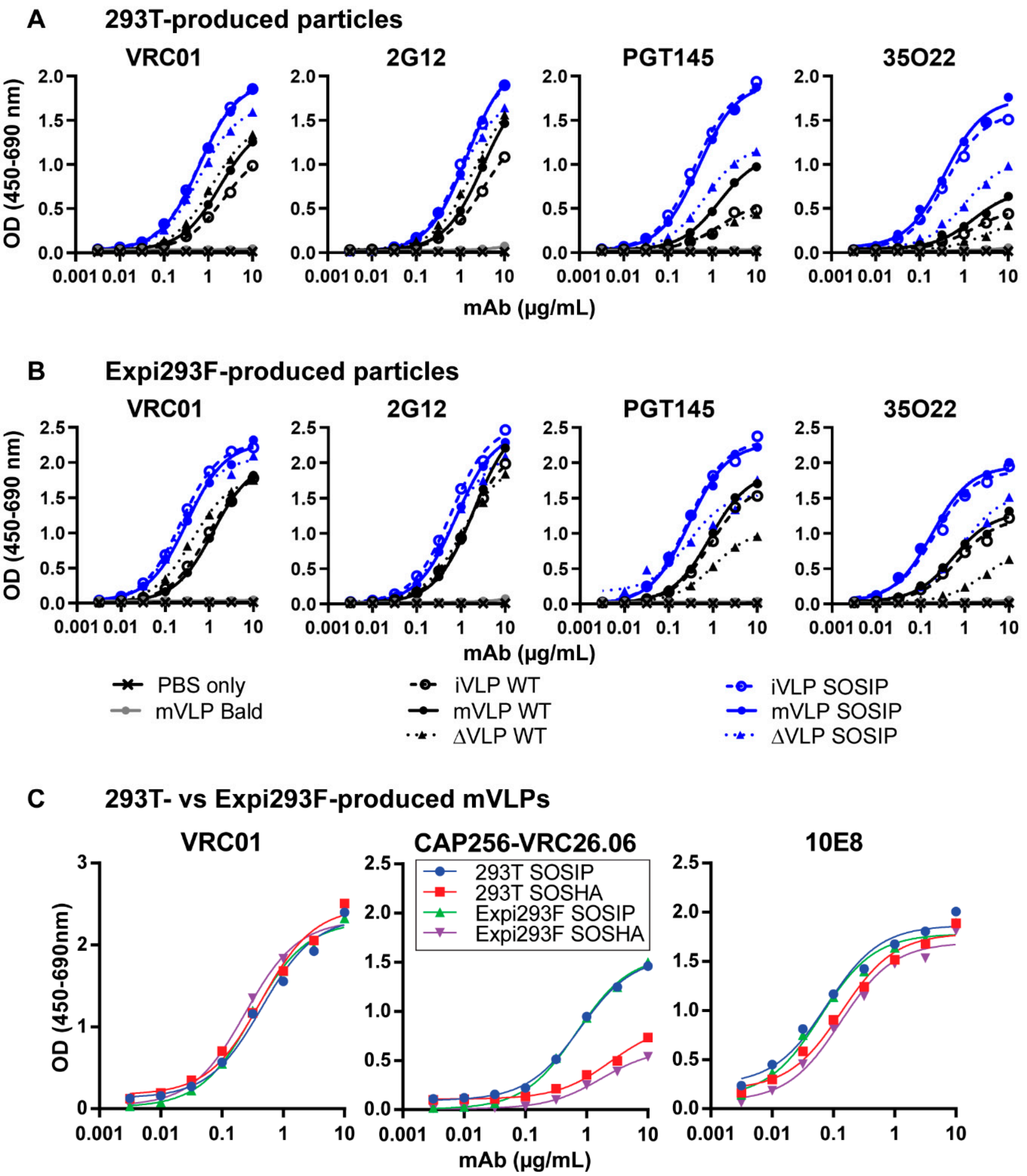

Figure S3. 293T- and Expi293F per-particle Env antigenicity, and comparison of 293T- versus Expi293F-produced particle-associated Env antigenicity. (**A, B**) ELISA binding curves of VRC01, 2G12, PGT145 and 35O22 to (**A**) 293T- or (**B**) Expi293F-produced iVLPs, mVLPs and ΔVLPs bearing wild type Env (WT) or SOSIP-modified Env (SOSIP). A buffer only control (PBS only) and mVLPΔenv particles (mVLP Bald) were also assayed. VLP ELISAs were performed using equal numbers of mVLPs as determined by p24 ELISA. iVLP loading was Env-equalized to the mVLP bearing the same Env produced in the same cell type, as determined by anti-gp120 Western blotting. ΔVLP loading was volume equalized to the higher volume loading from either the mVLP or iVLP sample bearing the same Env produced in the same cell type. (**C**) ELISA binding curves of VRC01, CAP256-VRC26.06 and 10E8 to mVLPs bearing SOSIP or SOSHA Env expressed from 293T or Expi293F cells. VLP ELISAs were performed with 200ng/well gp120 as determined by anti-gp120 Western blotting. For A–C, ELISA data is representative of results from two independent assays.

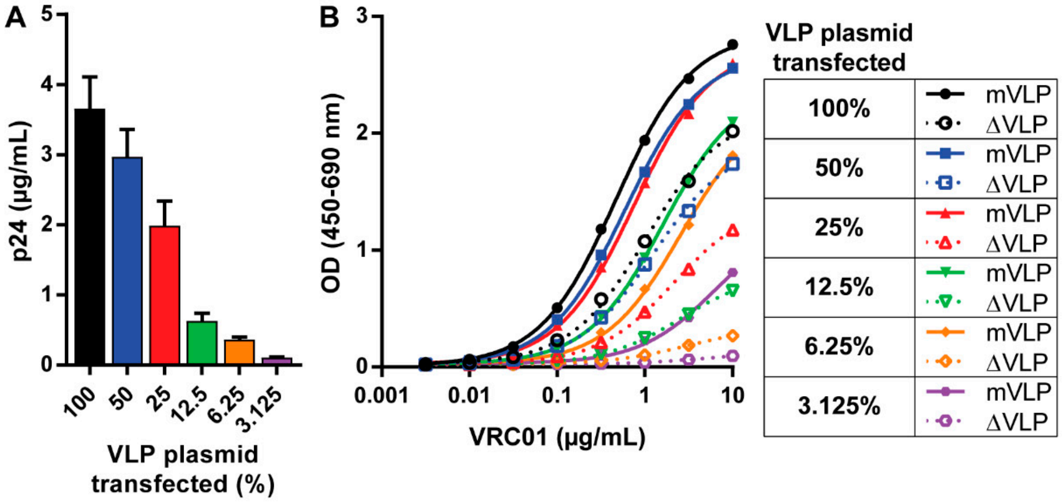

Figure S4. Assessing decreased transfection plasmid copy number for reduction of microvesicle- and exosome-associated Env from VLP preparations. Equal volumes of Expi293F cell suspensions were transfected with a 2-fold serial dilution (from 100% to 3.125% of total DNA) of mVLP SOSIP or ΔVLP SOSIP expression plasmids, which were DNA equalized with empty vector plasmid, before concentrating and resuspending VLPs in equal volumes. (**A**) The p24 concentration of the resultant mVLP suspensions as determined by p24 ELISA. Values represent the mean and SEM of within-assay duplicate measurements. (**B**) Representative VLP ELISA binding curves of VRC01 to mVLP and ΔVLPs each loaded with equal volume/well from two independently performed assays.

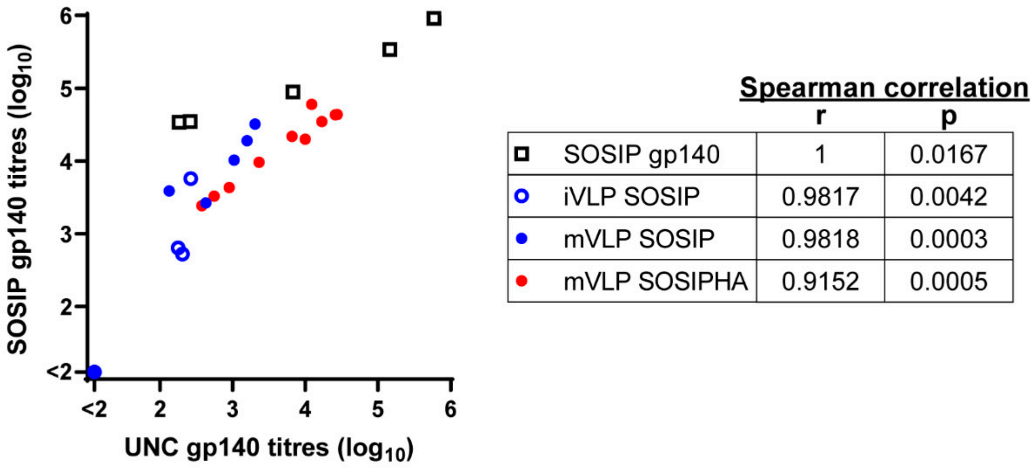

Figure S5. Correlation of UNC and SOSIP Env responses**.** Scatterplot of AD8 SOSIP gp140 versus AD8 UNC gp140 titers at week 16 from Figure 8B and C. Two-tailed Spearman correlations were determined for vaccination groups that had at least two animals with endpoint serum titers ≥2 for both SOSIP and UNC gp140 (SOSIP gp140, iVLP SOSIP, mVLP SOSIP and mVLP SOSIP). For each vaccination group the Spearman's rho (r) and P-value (p) are detailed in the inset.

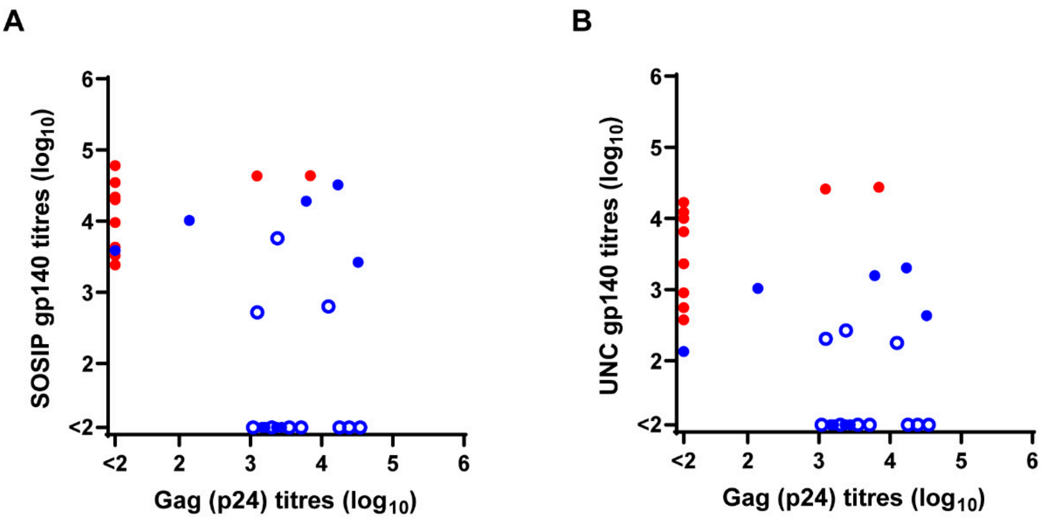

Spearman correlation (A)

	r	p
iVLP SOSIP	-0.2312	0.5306
mVLP SOSIP	0.2263	0.5557
mVLP SOSIPHA	0.5276	0.1333

Spearman correlation (B)

	r	p
iVLP SOSIP	-0.3057	0.4
mVLP SOSIP	0.3656	0.3304
mVLP SOSIPHA	0.7006	0.0222

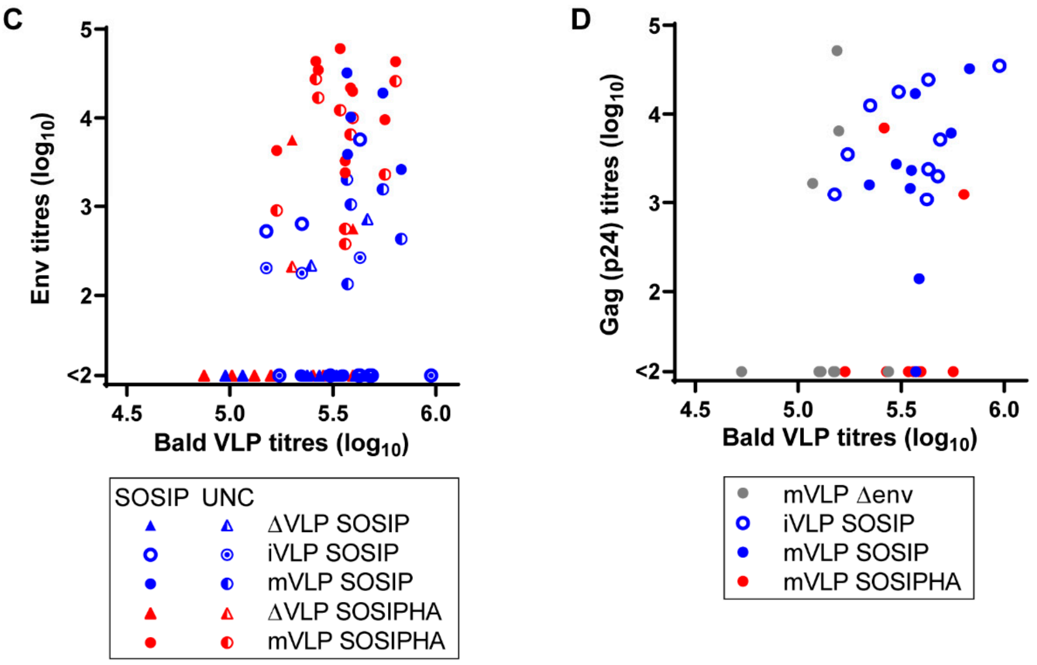

Figure S6. Correlation of Env, p24 and bald VLP mouse serum antibody responses. Scatterplots of iVLP and mVLP vaccination group 93TH253.3 p24 titers versus (**A**) AD8 SOSIP gp140 or (**B**) AD8 UNC gp140 titers at week 16. For (**A**) and (**B**), two-tailed Spearman correlations were determined for vaccination groups that received iVLPs or mVLPs bearing Env (iVLP SOSIP, mVLP SOSIP and mVLP SOSIP). For each vaccination group the Spearman's rho (r) and P-value (p) are detailed in the inset. (**C**) Scatterplot of ΔVLP, iVLP and mVLP Bald VLP titers versus AD8 SOSIP gp140 (closed circles) and AD8 UNC gp140 (open circles) titers at week 16. (**D**) Scatterplot of iVLP and mVLP Bald VLP titers versus 93TH253.3 p24 titers at week 16.

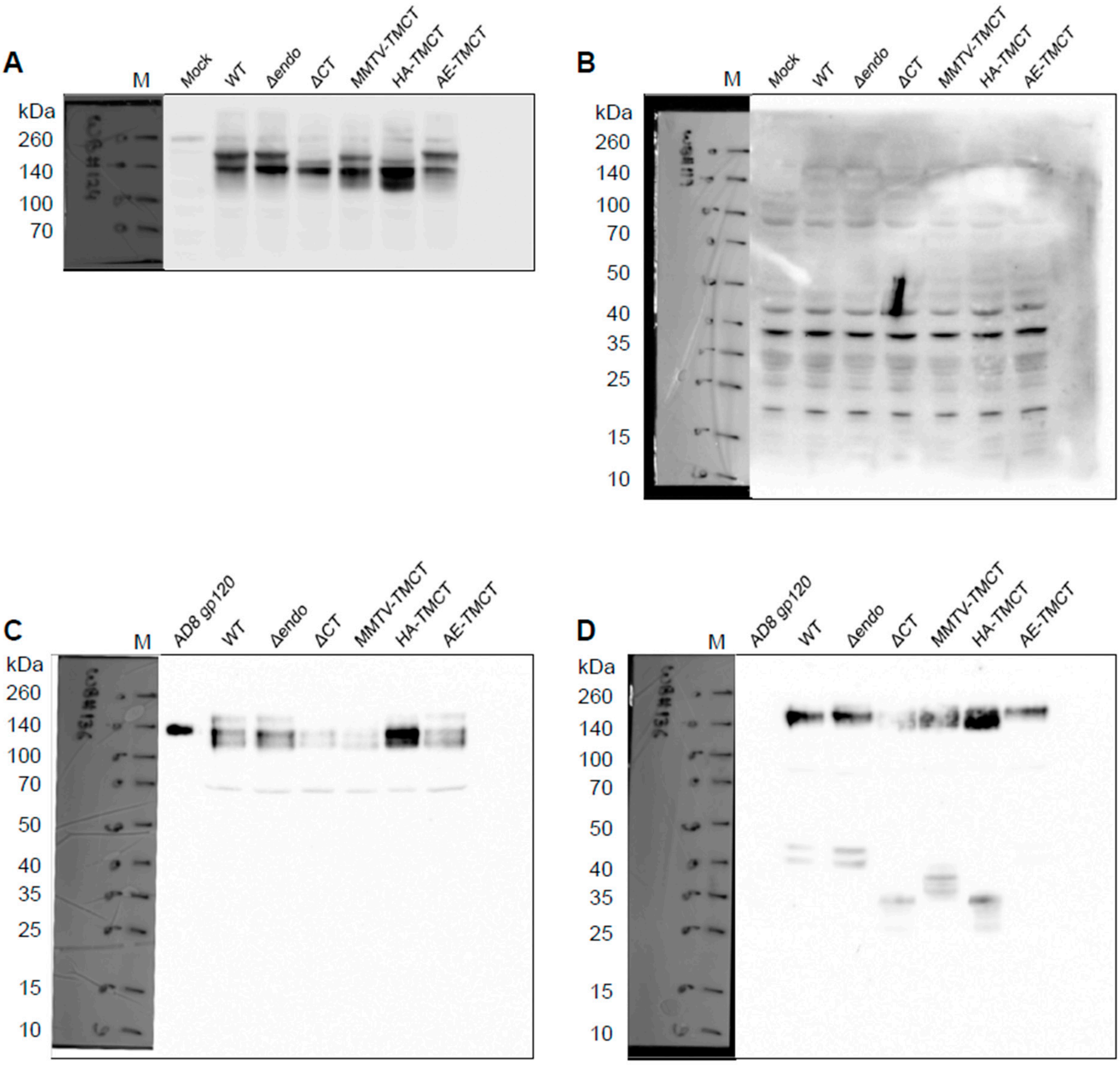

Figure S7. Uncropped images of Western blots from Figure 1B and 1C**.** (**A**) Upper anti-gp120 and (**B**) lower anti-GAPDH panels of Figure 1B. The blot shown in A does not represent the full length of the SDS-PAGE since the PVDF membrane (onto the which the SDS-PAGE was transferred) was cut into two pieces for subsequent Western blotting with different antibodies. The portion of the membrane with molecular weight markers from 50 to 10kDa is not shown here as the Western blotting performed was not reported in this manuscript. (**C**) Upper anti-gp120 and (**D**) lower anti-gp41 panels of Figure 1C. For all blots shown above, the bright light image of the protein size markers (lane "M") is overlaid on the left-hand side of the Western blot image. Protein size marker bands were marked with black pen prior to Western blotting for visibility. The identity of each marker band is indicated by their molecular weight value to the left.

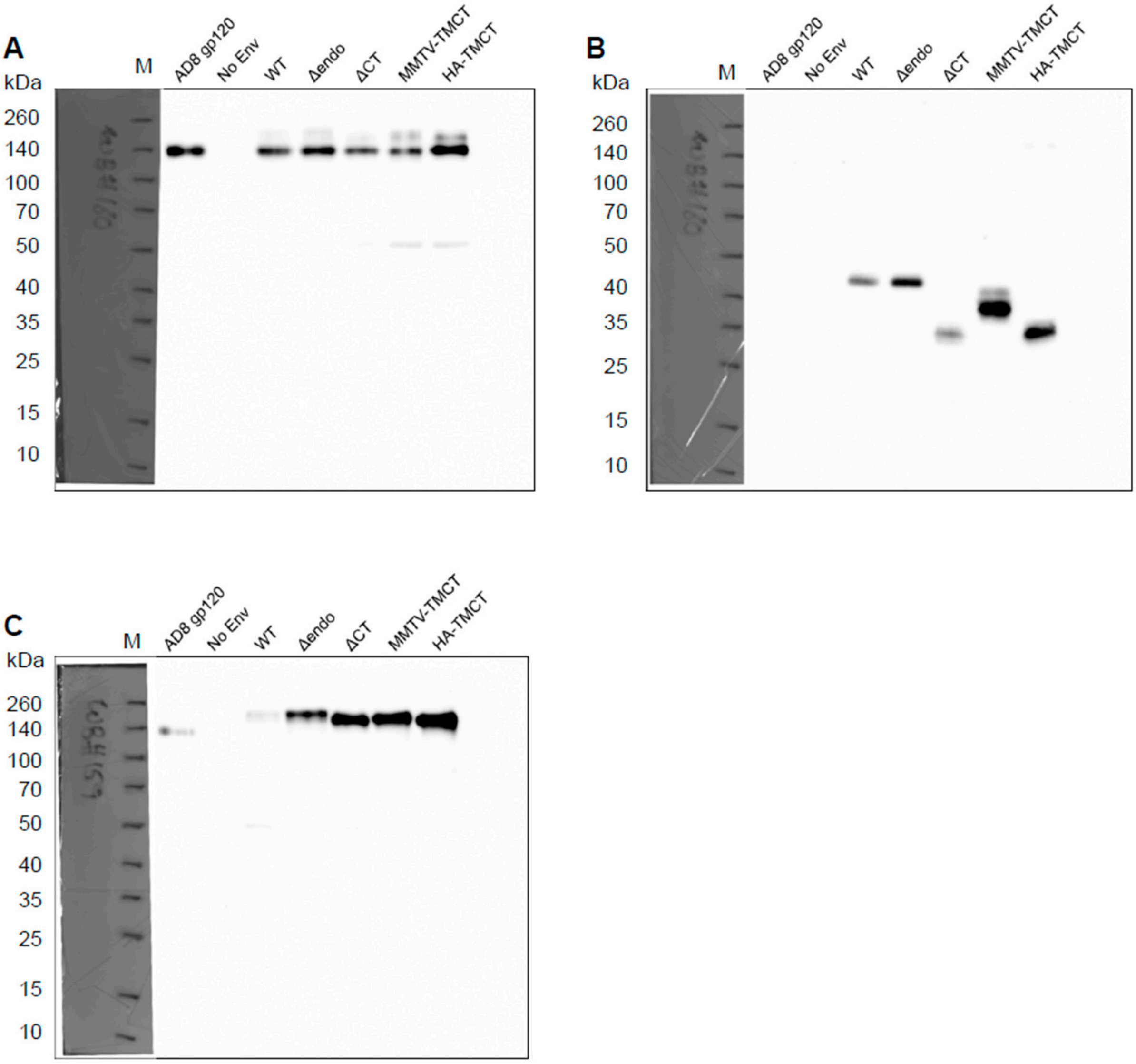

Figure S8. Uncropped images of Western blots from Figure 2B and 2C. (A) Upper anti-gp120 and (B) lower anti-gp41 panels of Figure 2B. (C) Upper anti-gp120 panel of Figure 2C. For all blots shown above, the bright light image of the protein size markers (lane "M") is overlaid on the left-hand side of the Western blot image. Protein size marker bands were marked with black pen prior to Western blotting for visibility. The identity of each marker band is indicated by their molecular weight value to the left.
